# Escaping and repairing behaviors of the termite *Odontotermes formosanus* (Blattodea: Termitidae) in response to disturbance

**DOI:** 10.7717/peerj.4513

**Published:** 2018-03-16

**Authors:** Hongpeng Xiong, Xuan Chen, Yuzhen Wen, Michael Layne, Zhaohui Sun, Tao Ma, Xiujun Wen, Cai Wang

**Affiliations:** 1 Guangdong Key Laboratory for Innovation Development and Utilization of Forest Plant Germplasm, College of Forestry and Landscape Architecture, South China Agricultural University, Guangzhou, Guangdong, China; 2 Department of Environmental Sciences, Louisiana State University, Baton Rouge, LA, United States of America

**Keywords:** Antipredator behavior, Collective behavior, “faster-is-faster” effect, Eusocial insect, Black-winged termite

## Abstract

The escaping behavior of termites has been documented under laboratory conditions; however, no study has been conducted in a field setting due to the difficulty of observing natural behaviors inside wood or structures (e.g., nests, tunnels, etc.). The black-winged termite, *Odontotermes formosanus* (Shiraki), is a subterranean macrotermitine species which builds extensive mud tubes on tree trunks. In the present study, 41 videos (totaling ∼2,700 min) were taken on 22 colonies/subcolonies of *O. formosanus* after their mud tubes were partially damaged by hand. In general, termites consistently demonstrated three phases of escape, including initiation (wandering near the mud-tube breach), individual escaping (single termites moving downward), and massive, unidirectional escaping flows (groups of termites moving downward). Downward moving and repairing were the dominant behavioral activities of individuals and were significantly more frequent than upward moving, turning/backward moving, or wandering. Interestingly, termites in escaping flows moved significantly faster than escaping individuals. Repairing behavior was observed shortly after the disturbance, and new mud tubes were preferentially constructed from the bottom up. When predators (i.e., ants) were present, however, termites stopped moving and quickly sealed the mud-tube openings by capping the broken ends. Our study provides an interesting example that documents an animal (besides humans) simultaneously carrying out pathway repairs and emergency evacuation without congestion.

## Introduction

The escaping behaviors of social and gregarious animals in response to emergency situations (e.g., the presence of predators or occurrence of disaster events) have received increasing attention in recent years (Lin et al., 2016; Lin et al., 2017). Solitary individuals commonly exhibit complicated escaping strategies such as turning or adjusting body orientations (Cooper Jr & Sherbrooke, 2016). However, grouped individuals have limited information due to high crowd density, which might cause physical competitions while escaping (Shiwakoti & Sarvi, 2013). For example, when escaping from the site of danger (e.g., burning buildings or flooding) through a narrow exit, humans and some other mammals (e.g., *Rattus norvegicus* Erxleben) have shown a “faster-is-slower” effect caused by the “selfish behaviors” (i.e., trampling, crushing, pushing, etc.) of individuals that decreased escaping speed and evacuation efficiency (Helbing, Farkas & Vicsek, 2000; Helbing, Johansson & Al-Abideen, 2007; Shiwakoti, Sarvi & Burd, 2014).

Studies on social insects, such as ants, have shown different behavioral responses from vertebrates under rapid-group-escape conditions (Altshuler et al., 2005; Boari, Josens & Parisi, 2013; Parisi, Soria & Josens, 2015; Wang, Lv & Song, 2015; Wang & Song, 2016). When escaping from a disturbed arena through an exit or passageway, ants exhibited no “selfish evacuation behavior” that could cause congestion. This “faster-is-faster” effect in ants can be attributed to several strategies. For example, the panicked ants moved in several directions so no sharp increase in ant density occurred near the exit; also, ants were uniformly distributed and there was no “high contact between ants” while exiting (Boari, Josens & Parisi, 2013; Parisi, Soria & Josens, 2015). Studies on a large ant species, *Camponotus japonicus* Mayr, showed that ants divided themselves into several groups near the exit to reduce density under emergency conditions (Wang, Lv & Song, 2015). When passing through a straight passageway, the evacuation speed of *C. japonicas* was unexpectedly constant with increasing ant density (Wang & Song, 2016).

Termites are another group of eusocial insects that live at high density, but their escaping behavior has received less attention compared to ants. The termite escaping process takes place inside wood and structures (e.g., nests, tunnels, mud tubes, etc.), which makes observation difficult under laboratory or natural conditions. Several studies that examined the response of termites to a disturbance (e.g., vibrational stimuli such as knocking) have been conducted on arenas containing food and termite foragers (Hu, Appel & Traniello, 2003; Schwinghammer & Houseman, 2006; Gautam & Henderson, 2012). These studies showed that termites immediately escaped from the source of disturbance to other containers (without food) through connecting tubes, but they returned after a short period (within several seconds to minutes). Based on these observations, researchers concluded that termites would not abandon food sources “for extensive periods of time as a result of mechanical disturbances” (Gautam & Henderson, 2012). However, only small groups of termites (100–200 individuals) were tested in these laboratory studies, which might differ from field conditions since a mature colony of subterranean termites may contain thousands to millions of individuals (Su, Scheffrahn & Ban, 1995).

Using a special experimental setting, Wang et al. (2016) reported that workers of *Coptotermes formosanus* Shiraki escaped along the wall of a Petri dish after a disturbance was created by knocking, and the unidirectional escaping flow (either clockwise or counter-clockwise) lasted for long periods (>40 min). During the escaping process, little congestion was observed, probably because most worker termites followed each other, whereas only a few workers reversed or moved backward against the escaping flow. Wang et al. (2016) was the first reported study to document the behavioral patterns of escaping termites. However, it was still a laboratory observation which may differ from a real-world situation. A few studies have focused on the behavioral responses of termites to damaged foraging galleries and mounds in the field. For example, Pequeno (2017) reported that after a breach (1-cm long) was opened in the foraging gallery, some soldiers (ranging from 1 to 12) of *Nasutitermes corniger* (Motschulsky) patrolled in the open area (lasting from 2 to 292 s). Zachariah et al. (2017) reported that workers of *Odontotermes obesus* (Rambur) repaired the broken mound by attaching various materials (e.g., soil particles and grass fragments of different sizes) along the entire circumference of the breach.

The black-winged termite, *Odontotermes formosanus* (Shiraki), is a severe forestry pest distributed in China and Southeast Asia. This species is known to build subterranean nests and construct extensive mud tubes and/or shelters on tree trunks, which favor the consumption of bark and wood (Cheng et al., 2007; Appel et al., 2012). Our preliminary observations showed a long-lasting and massive termite exodus from the tree crown to underground tunnels as well as repairing behaviors when the mud tube was damaged. This phenomenon provides a valuable opportunity to understand escaping and repairing behaviors of termites under natural conditions. In the present study, we aimed to: (1) determine how groups of termites organize while escaping from and repairing broken mud tubes, and (2) describe the behavioral activities of termites during these processes.

## Materials and Methods

### Study site

The field study was conducted from 5 September to 4 November 2016 on the campus of South China Agricultural University, Guangzhou, China (locations are shown in Table S1). We searched mud tubes constructed by *O. formosanus* on tree trunks, and checked for activity by creating a small hole (∼1–2 cm^2^) on the mud tube 2–3 days before the test. Mud tubes which showed termite activity (moving and repairing) were used for this study. Only one mud tube per tree was selected in this study. However, it is worth noting that some mud tubes might belong to the same termite colony because several trees were not far from each other (<100 m). Altogether, 22 colony/sub-colony groups of *O. formosanus* were tested in the present study.

### Mud-tube damage and video recording

**Figure 1:**
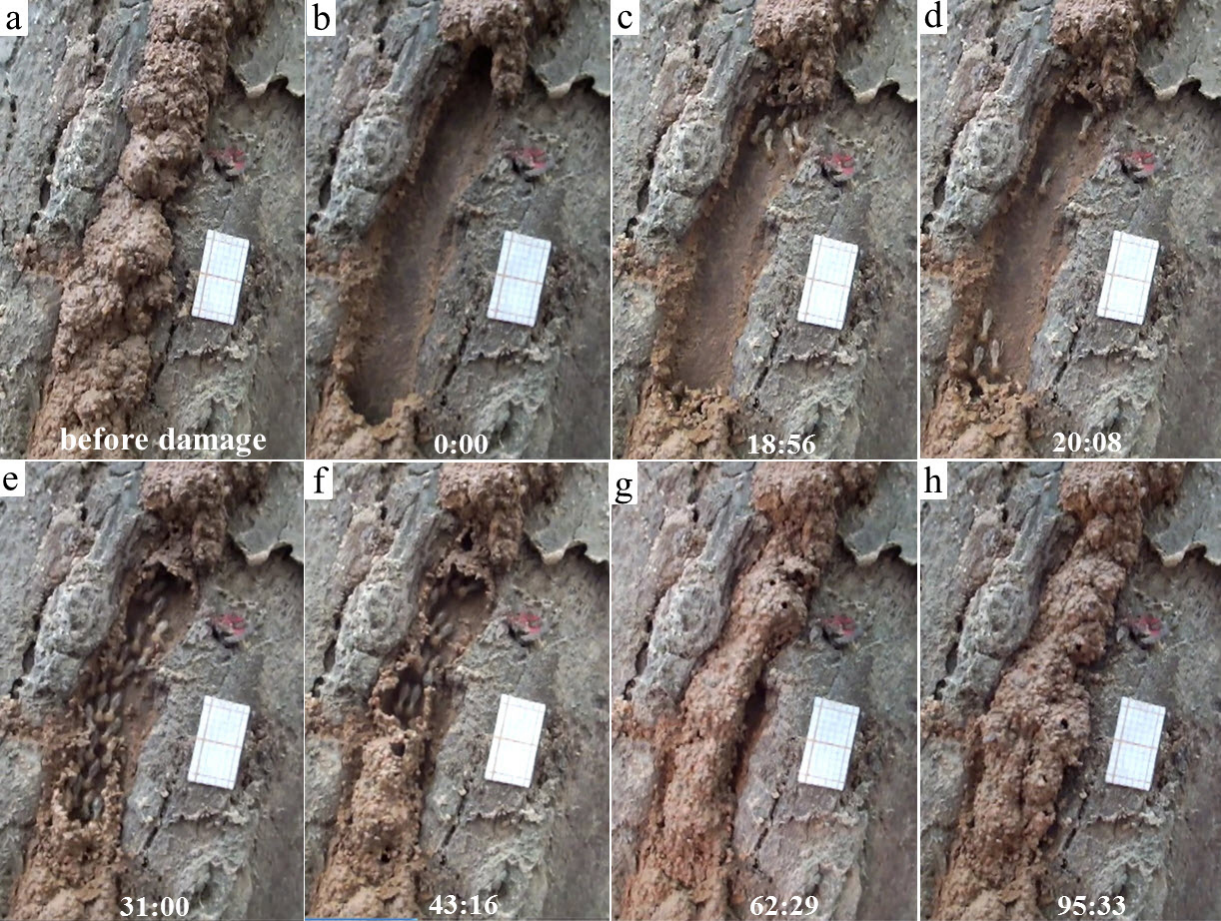
Repairing and escaping behaviors of *Odontotermes formosanus* individuals. In this observation, the mud tube was shown before (A) and right after (B) the damage. The initiation phase (single or groups of termites wandered near the mud tube breach) was observed at 18:56 (C), individual escaping (individual termites moving downward through the exposed pathway) was observed at 20:08 (D), and escaping flow (groups of termites moving downward through the exposed pathway) was observed at 31:00 (E). The mud tube was closed preferentially from bottom up (as shown at 43:16) (F), and at 62:29 the tube was closed (G). Subsequent modifications were observed when termites added more soil particles to broaden the repaired section (as shown at 95:33) (H). Before the test, a piece of graph paper (1.2 by 2.2 cm, 10 lines per centimeter) was pasted near the mud tube with its long side parallel to the mud tube. This video was taken on 14 September 2016.

Tests were conducted between 8:00 and 18:00. Tree species, diameter (measured at the height of mud-tube damage), location coordinates, and weather conditions were recorded (Table S1). Before each test, a piece of graph paper (1.2 by 2.2 cm, 10 lines per centimeter) was pasted near the mud tube with its long side parallel to the mud tube for the measurement of repairing areas (Fig. 1). The mud tube (forming an upside down “U” shape in cross section) was removed from a short fragment (56.9 ± 8.0 mm [mean ± SD, *n* = 41] in length) by hand, and the responses of termites were videotaped until the mud tube was repaired and closed, or until 2 h had passed without the tube being closed. In the latter case, the repair status of the broken mud tubes was checked the next day.

Previous laboratory studies found that while termites immediately escaped after a disturbance, foraging sites were not abandoned (Hu, Appel & Traniello, 2003; Schwinghammer & Houseman, 2006; Gautam & Henderson, 2012). To verify this in the field, we conducted a second test for each previously selected colony/subcolony of *O. formosanus* within 2–7 days after the initial disturbance. We hypothesized that termites would not abandon the mud tube after the first disturbance test, and therefore their activities can be detected in the second test. The mud tubes of three termite groups were completely destroyed by a rainstorm during the 2–7 day interval between tests. Therefore, 41 videos (total length ∼2,700 min) were taken and used to observe the escaping and repairing behaviors of termites.

### Behavioral activity of individual termites

In the present study, five behavioral activities of termites were identified after the disturbance: (i) repairing—termites rebuilt the damaged mud tube by attaching soil particles onto the ends of the breached tubes or along the sides of the exposed pathway; (ii) wandering—termites stayed close or slowly moved (stepped forward and then back) near the mud-tube breach and did not stray far from the breach; (iii) escaping—termites moved downward through the exposed pathway; (iv) upward moving—termites traveled from bottom to the top (the opposite direction of escaping) and passed through the exposed pathway; and (v) turning/backward moving—termites changed direction or walked backward while escaping and did not pass through the exposed pathway. Wandering termites only stayed close or moved slowly near the openings of the mud-tube breach, so that they were distinct from termites that exhibited turning/backward moving. Termite repairing, escaping, upward moving, and turning/backward moving behaviors had been previously identified and described (Wang et al., 2016; Zachariah et al., 2017), whereas wandering was first identified in the present study.

For Type I and II videos (see ‘Results’), the number of termites that exhibited each behavioral activity (wandering, escaping, upward moving, turning/backward moving, and repairing) was counted within a 15-s period every 2 min until the mud tube was closed or until 2 h had passed. The five behavioral activities provided an exhaustive list so that all termites could be visually categorized. Here we did not differentiate soldier and worker *O. formosanus* because only a few foragers (<1%) are soldiers (Soleymaninejadian et al., 2014). Also, no new behaviors (such as patrolling observed in *N. corniger* (Pequeno, 2017) were observed in soldier *O. formosanus* compared to workers once the mud tube was damaged.

### Escaping speed of termites

Ten Type I videos (see ‘Results’) were randomly selected for escaping speed measurement. Since the escaping processes can be divided into the individual escaping phase and escaping-flow phase (see ‘Results’), speed was measured separately for each phase. Ten termites that exhibited escaping behavior were randomly selected within the individual escaping phase of each video for speed measurement. We also measured the speed of 10 individual termites during the escaping flow phase, which was separated into three time periods (5–6 min after the escaping flow was formed, in the middle of the escaping flow duration, and 5–6 min before the escaping flow ended). The speed of these termites was measured by dividing the length of exposed pathway by the passing time of termites.

### Mud-tube repairing and subsequent modifications

ImageJ (National Institutes of Health, Bethesda, MD, USA) was used to measure the area of the mud tube that was repaired from the bottom to top, top to bottom, or along the sides of the exposed pathway for every 10-min period until the mud tube was closed (closure was determined when the whole pathway was covered by the mud tube, though one or several small holes [<5 mm in diameter] may be observed on the repaired mud tube). Only Type I videos (see ‘Results’) were analyzed. Since most mud tubes were closed in <60 min, the repaired area was measured only during the 1-h period. Any subsequent modifications (after the mud tube was closed) were also observed and recorded (Fig. 1).

### Data analysis

Mixed-effect linear models were built to assess differences of termite escaping speed among different phases (periods), treating phase (periods) as a fixed factor and video as a random factor. Tukey’s Honestly Significant Difference (HSD) tests were conducted for pairwise comparisons.

Videos were divided into three groups due to (1) high variability of tube-closing time and (2) previous observations showing that the observed number of termites was a function of tube-closing time: Group 1 (six videos): videos ended within 22 min; group 2 (15 videos): videos ended between 24 and 40 min; and group 3 (nine videos): videos ended after more than 42 min. Models were built separately for each group in the following analyses.

Generalized linear mixed-effect models were applied to study (1) the number of termites performing each behavioral activity with behavior, time, and behavior × time as fixed factors and video as random factor, and (2) the differences of repairing area from different directions with direction, time, and direction × time as fixed factors and video as a random factor. Pairwise comparisons using Tukey’s HSD tests were performed post hoc to study the differences of repairing area and number of termites demonstrating different behavioral activities at each time period.

The analysis was conducted with R packages “lme4”, “lmerTest”, and “lsmeans” in the statistical program “R 3.3.2” (R Development Core Team, 2016). Significance levels for all analysis were determined at *α* = 0.05.

## Results

### Process and categories of termite responses

In general, termite activity was observed in both disturbance tests for each colony/subcolony group of termites, except for three groups where the mud tubes were destroyed by a rainstorm during the 2–7 day interval (Table S2). In most videos, the escaping process of termites could be divided into three phases: (i) initiation phase—a single or groups of termites wandered near the mud-tube breach; (ii) individual escaping phase—a single or a few termites moved downward through the exposed pathway; and (iii) escaping flow phase—groups of termites moved downward through the exposed pathway (Fig. 1).

Based on the behavioral patterns of repairing and escaping and the presence of predators (ants), termite responses to mud-tube damage were divided into four exclusive categories (Table S2): Type I (31 videos)—all three phases of escaping were observed, and the mud tube was closed within the 2-h recorded period; Type II (6 videos)—groups of predators (ants) were observed, and the mud tube was closed within 2 h in only 1 video; Type III (3 videos)—the mud tube was closed within the 2-h recorded period, but escaping flow was not formed; and Type IV (1 video)—the mud tube was not closed at 2 h, and no predators were observed during recording (so that it could be distinct from Type II).

### Temporal patterns of termite activity at the site of the mud-tube damage

**Figure 2:**
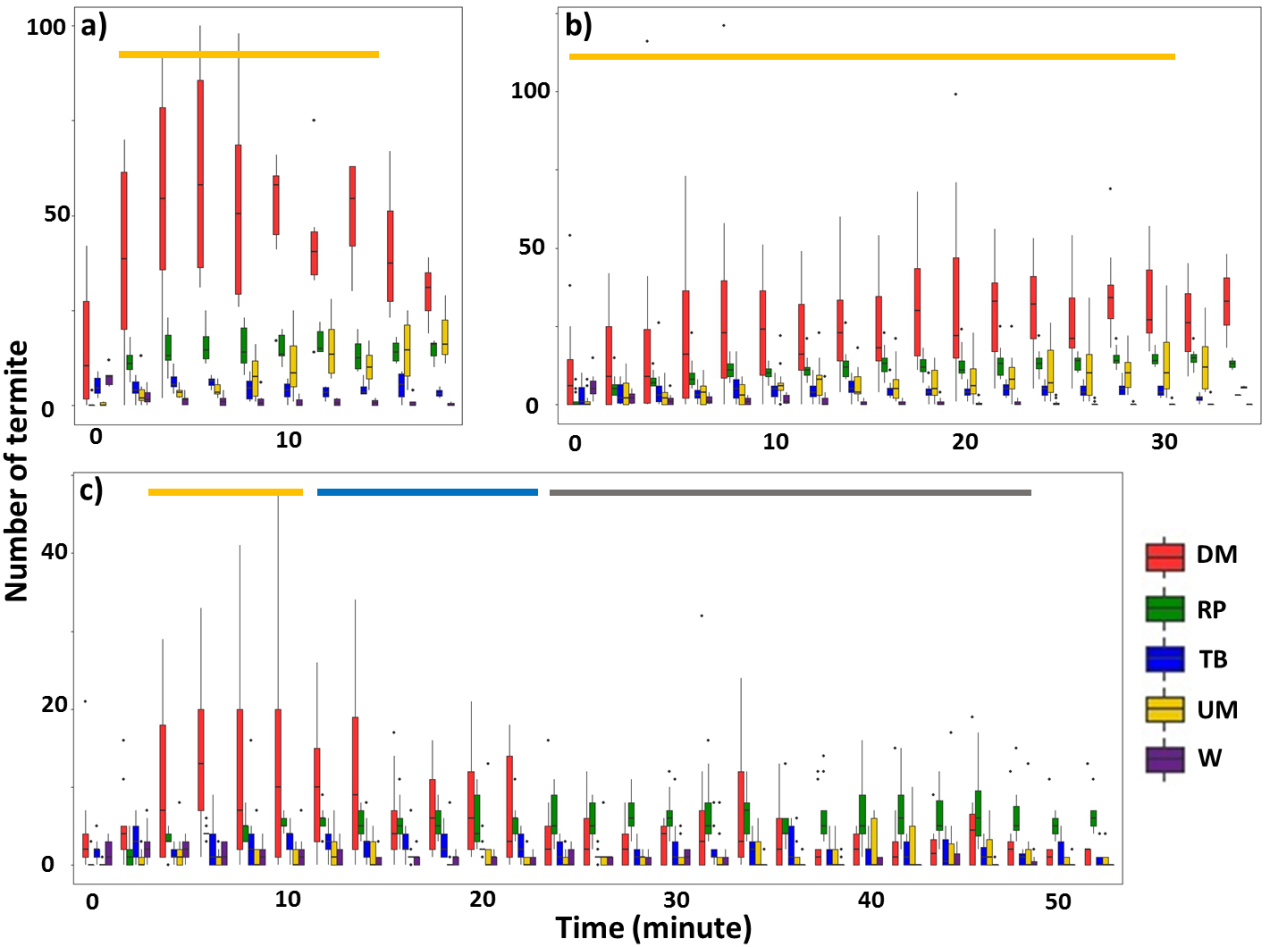
Number of termites performing different behavioral activities within a 15-s period for 2-min intervals following removal of an ∼5 cm section of the mud tube, for trials in which the damaged mud tube was completely repaired within 22 min (six videos) (A), between 24 and 40 min (15 videos) (B), and more than 42 min (nine videos) (C). Data show the number of termites that exhibited downward moving (DM, red), repairing (RP, green), turning/backward moving (TB, blue), upward moving (UM, yellow), and wandering (W, purple). Boxes show the 25th percentile, 50th percentile (median), 75th percentile, and the maximum length of each whisker is 1.5-fold the interquartile range. Dots indicate outliers. Generalized linear mixed-effect models were used to compare the number of termite individuals that showed different behaviors, and Tukey’s Honestly Significant Difference tests were performed for post-hoc multiple comparisons at each time period. The time period covered by the golden bar indicates that the number of downward moving termites was significantly higher than the number of termites performing all other behavioral activities. The blue bar shows the time period during which the number of downward-moving termites was significantly higher than the number of termites performing all other behaviors except repairing. The grey bar shows the time period during which the number of repairing termites was significantly higher than the number of termites performing upward moving, turning/backward moving, and wandering. Note that (A–C) differ in the scale of the *y*-axis.

For Type I videos, when the mud tube was closed within 40 min (Groups 1 and 2), downward escaping termites were significantly more frequent than termites showing other behavioral activities (Figs. 2A, 2B; Table 1; Tables S3, S4). For mud tubes that were closed after 40 min (Group 3), downward escaping termites were significantly more frequent than termites performing other behaviors between 4 and 10 min. From 12–22 min, downward escaping termites tended to be more common than upward moving, wandering, and turning/backward moving termites, but were not significantly more common than termites showing repairing behavior. After 24 min, termites demonstrating repairing behavior tended to be more common than upward moving, wandering, and turning/backward moving termites (Fig. 2C).

**Table 1:** Summary of generalized linear mixed-effect models comparing the number of termite (*Odontotermes formosanus*) individuals that performed downward escaping with the number performing other behaviors after the mud tube had been damaged. Results are presented only for the comparison of number of termite individuals performing behavior; effects of time and the interaction of time with behavior are not presented.

	Estimate	SE	*Z* value	*P*
(A) Damaged mud tubes were closed within 20 min				
Intercept	2.669	0.205	13.055	<0.0001
Repairing	−3.103	0.545	−5.690	<0.0001
Turning/backward	−1.055	0.288	−3.660	0.0003
Upward moving	−3.838	0.764	−5.022	<0.0001
Wandering	−0.745	0.295	−2.525	0.0116
(B) Damaged mud tubes were closed between 20 and 40 min				
Intercept	2.387	0.132	18.047	<0.0001
Repairing	−2.116	0.255	−8.295	<0.0001
Turning/backward	−1.358	0.203	−6.682	<0.0001
Upward moving	−2.359	0.308	−7.662	<0.0001
Wandering	−0.763	0.202	−3.772	0.0002
(C) Damaged mud tubes were closed beyond 40 min				
Intercept	1.186	0.315	3.762	0.0002
Repairing	−2.350	0.628	−3.745	0.0002
Turning/backward	−0.745	0.320	−2.331	0.0198
Upward moving	−3.391	0.754	−4.501	<0.0001
Wandering	−0.753	0.422	−1.784	0.0745

**Figure 3:**
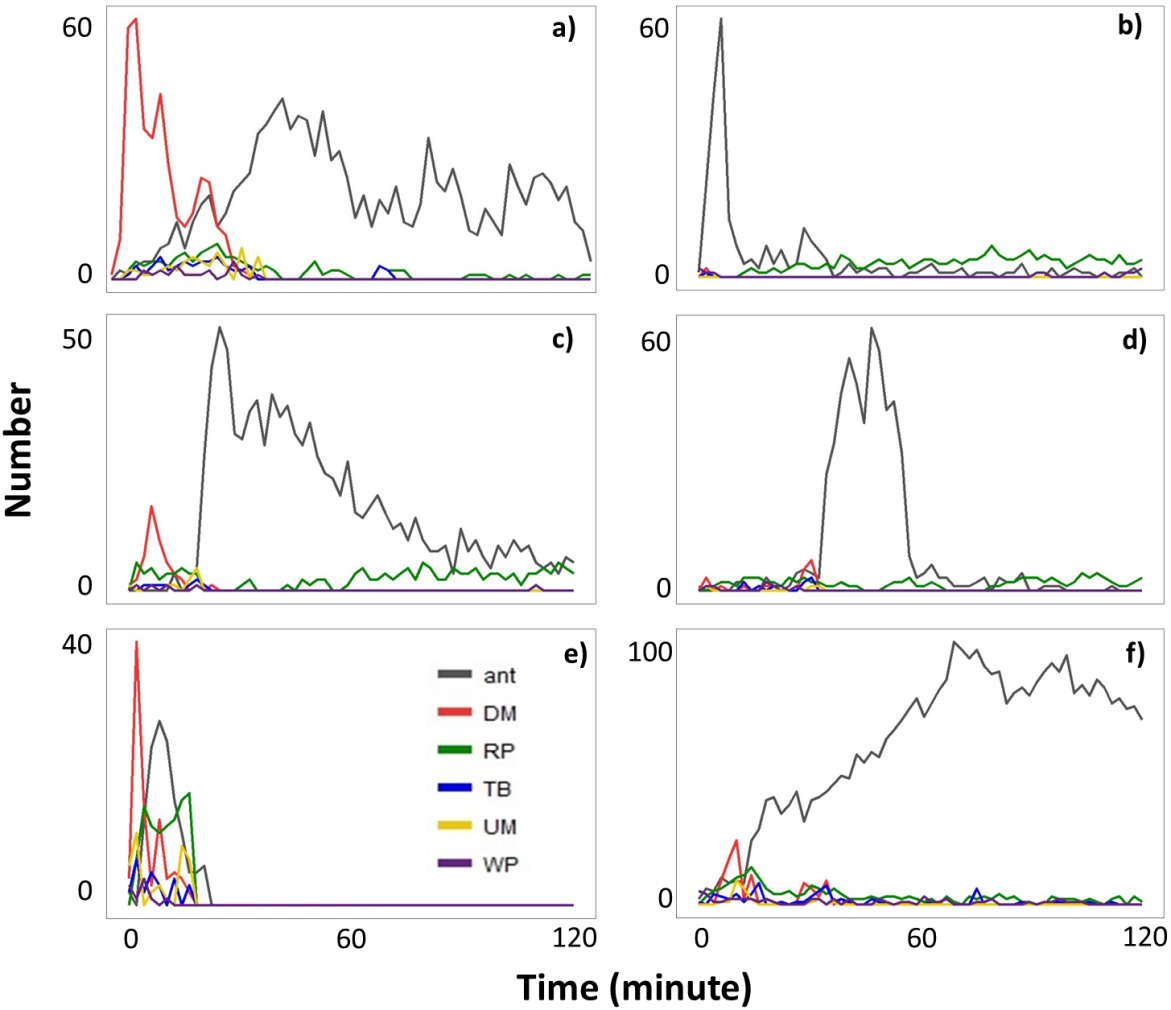
The number of ants and the number of termites performing different behavioral activities within a 15-s period every 2-min following removal of a ∼5 cm section of the mud tube in six Type II videos (A–F). Data are shown separately for ants (grey), and termites that exhibited downward moving (DM, red), repairing (RP, green), turning/backward moving (TB, blue), upward moving (UM, yellow), and wandering (W, purple). Note that (A–F) differ in the scale of the *y*-axis.

**Figure 4:**
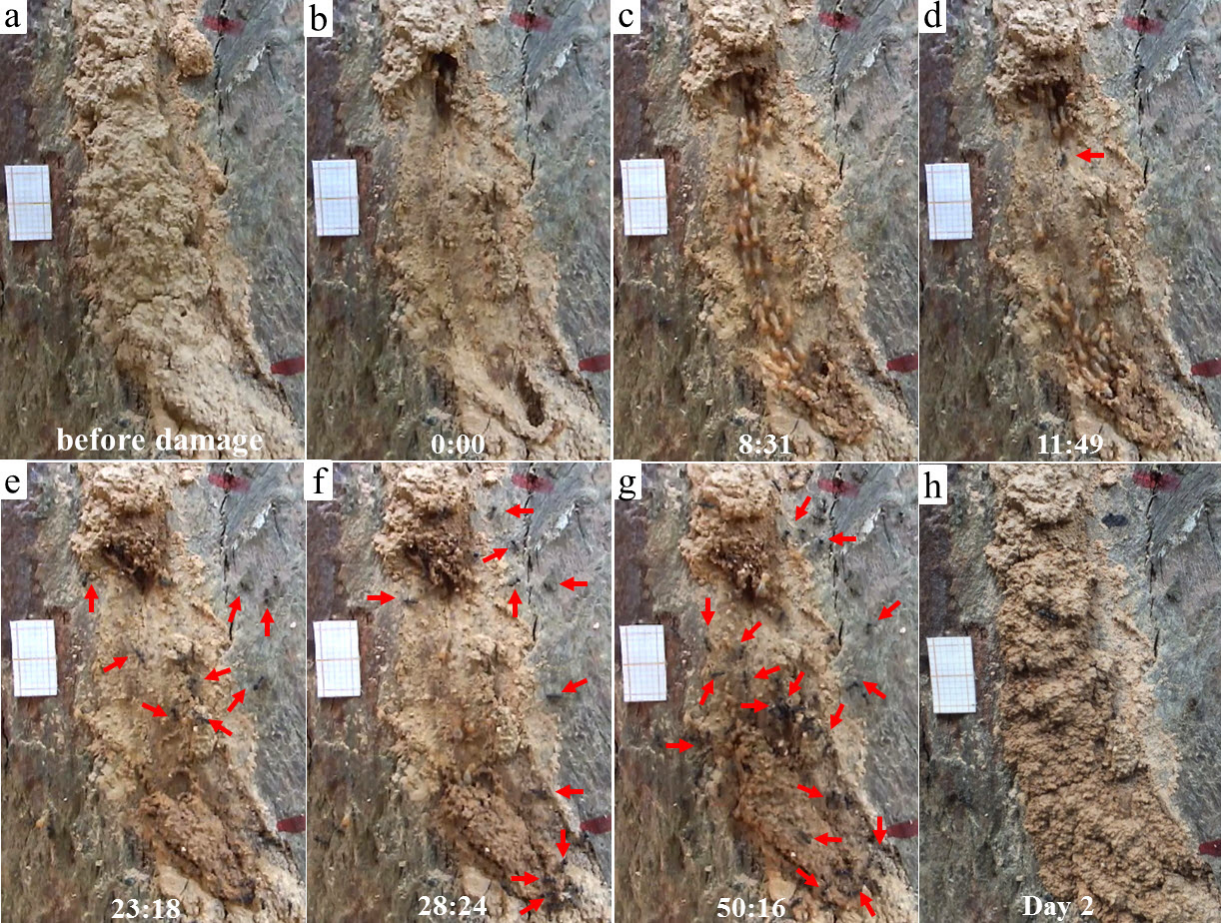
Behaviors of individual termites in response to the presence of ants. In this observation, the mud tube was shown before (A) and right after (B) the damage. Escaping flow was observed at 8:31 (C), but after predators (ants) were recruited at 11:49, termites immediately stopped moving on the exposed pathway (D), and then sealed the mud-tube openings by capping the broken ends so that the mud tube was not connected (as shown at 23:18) (E). After several minutes, termites made a hole on the seal and added particles to extend the mud tube (as shown at 28:24) (F). But when ants repeatedly attacked the termites, the mud tube was sealed again (as shown at 50:16) (G). The mud tube was eventually closed by the second day (H). The red arrows indicates ants. This video was taken on 27 October 2016.

When ants were recruited (Type II videos), termites immediately stopped moving across the exposed pathway (Fig. 3). Termites then sealed the mud-tube openings by capping the broken ends so that the mud tube was not connected. After several minutes, termites made a small hole on the seal and added soil particles to extend the mud tube. However, if ants continually attacked termites, the hole was sealed again. Only one repaired mud tube was closed within the 2-h period. Although the other five tubes were not closed within 2 h, they were eventually closed by the second day (Fig. 4).

### Escaping speed of termites

**Figure 5:**
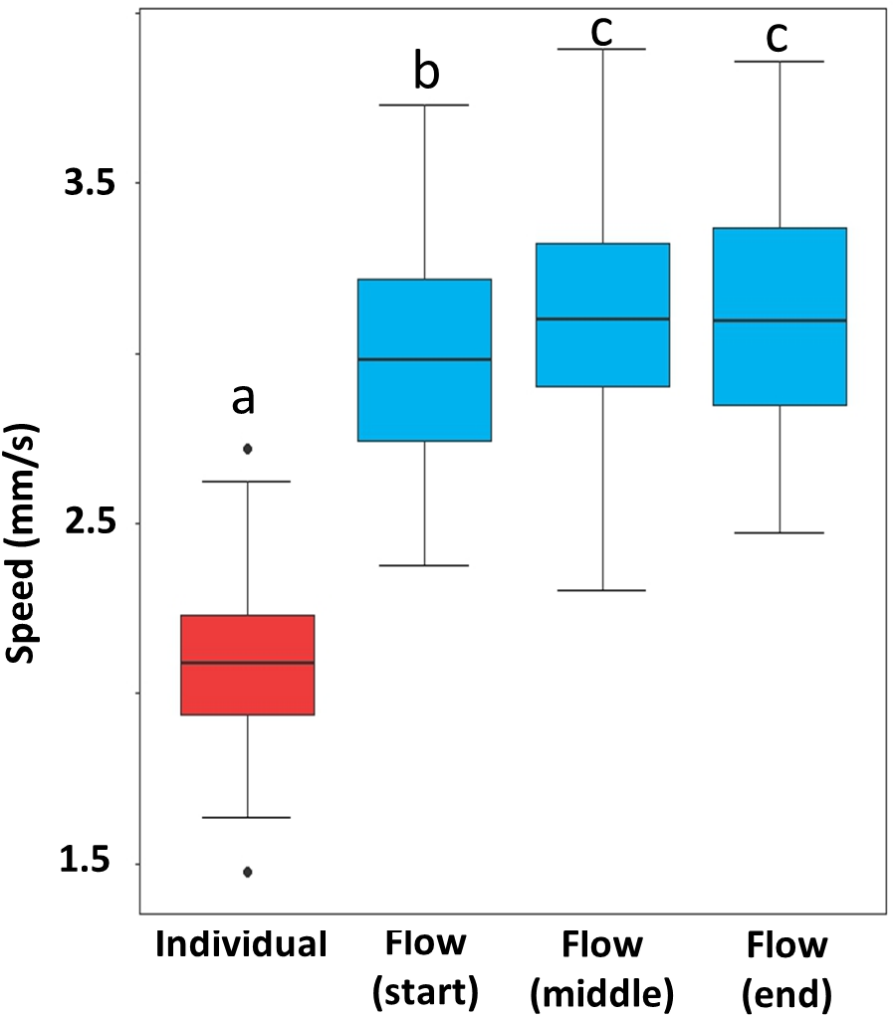
Speed of escaping termites in the individual escaping phase (red) and three periods of the escaping flow phase (blue). Box and whisker plots are based on 10 individual measurements on each phase (period) of each of the 10 video recordings (*n* = 100). Boxes show the 25th percentile, 50th percentile (median), 75th percentile, and the maximum length of each whisker is 1.5-fold the interquartile range. Dots indicate outliers. Different letters represent significant differences (*P* < 0.05), based on mixed-effect linear models.

Termites in escaping flows moved significantly faster than those that escaped individually. After escaping flow formed, termite speed varied by stage. Flow at the starting stage was slower than the two later stages (Fig. 5, Table S5).

### Mud-tube repairing and subsequent modifications

**Figure 6:**
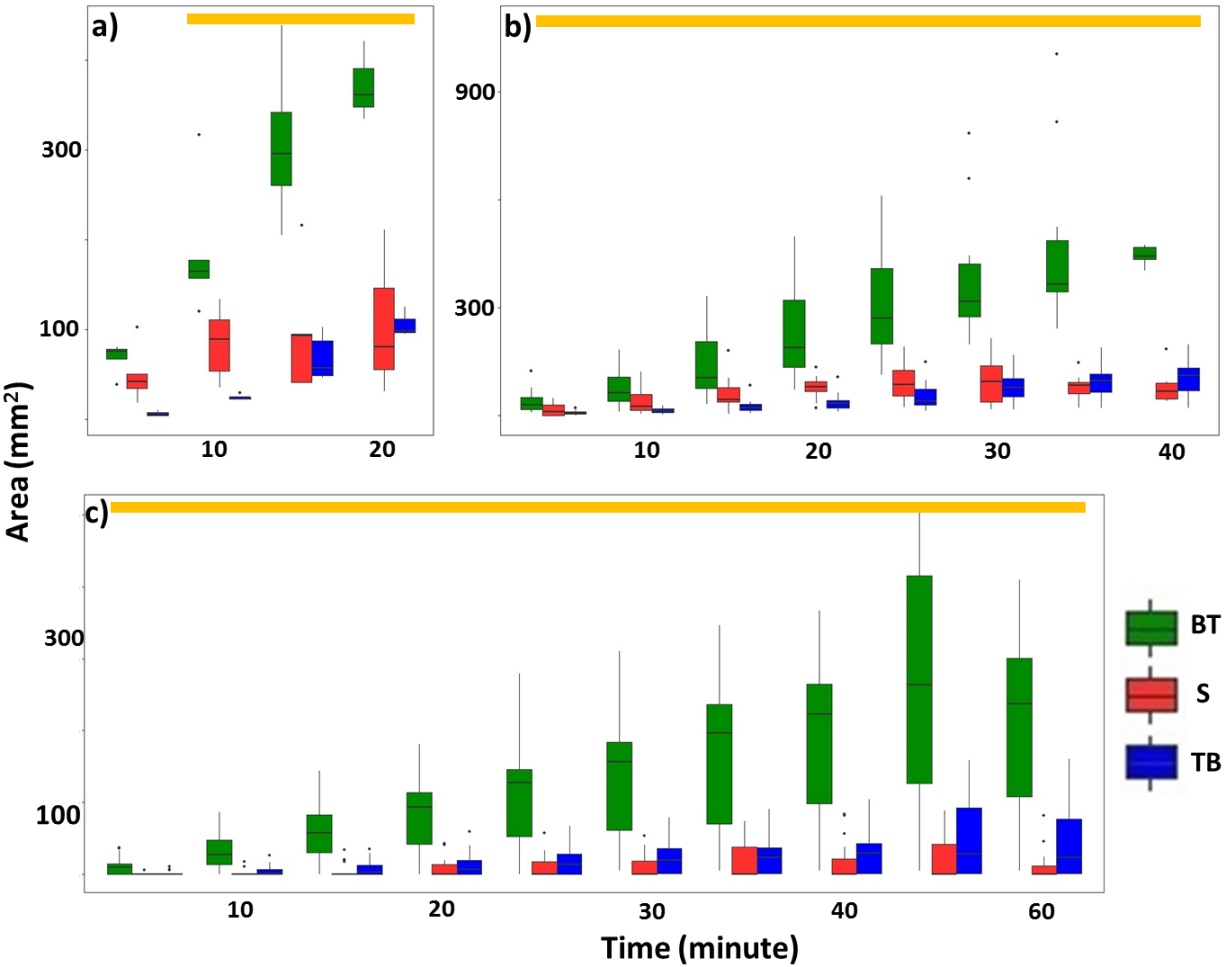
Total area (mm^2^) of mud tubes repaired by termites at 5-min intervals following removal of an ∼5 cm section of the mud tube, for trials in which the damaged mud tube was completely repaired within 22 min (six videos) (A), between 24 and 40 min (15 videos) (B), and more than 42 min (nine videos) (C). Data are shown separately for mud tubes repaired from bottom to top (BT, green), sides (S, red), and top to bottom (TB, blue). Boxes show the 25th percentile, 50th percentile (median), 75th percentile, and the maximum length of each whisker is 1.5-fold the interquartile range. Dots indicate outliers. Generalized linear mixed-effect models were used to compare the repaired area from different directions, and Tukey’s Honestly Significant Difference tests were performed for post-hoc multiple comparisons at each time period. Time periods covered by a golden bar at the top of (A–C) indicate that the area repaired from the bottom to top was significantly greater than from other directions.

Overall, termites consistently repaired significantly larger areas of mud tubes from the bottom to top rather than from the top to bottom or along the sides of the exposed pathway (Fig. 6; Table 2; Tables S6, S7). After the mud tubes were closed, termites continually added more materials to broaden the recently repaired sections (Fig. 1).

**Table 2:** Summary of generalized linear mixed-effect models comparing the repairing area constructed from bottom to top with other directions of the termite (*Odontotermes formosanus*) after the mud tube had been damaged. Results are presented only for the comparison of the area of repairing direction; effects of time and the interaction of time with repairing direction are not presented.

	Estimate	SE	*t* value	*P*
(A) Damaged mud tubes were closed within 20 min				
Intercept	4.200	0.184	22.878	<0.0001
Top to bottom	−2.145	1.590	−1.349	0.1770
Side	−0.744	0.425	−1.751	0.0800
(B) Damaged mud tubes were closed between 20 and 40 min				
Intercept	3.628	0.156	23.205	<0.0001
Top to bottom	−1.750	0.664	−2.635	0.0084
Side	−0.920	0.340	−2.705	0.0068
(C) Damaged mud tubes were closed beyond 40 min				
Intercept	0.038	0.008	4.761	<0.0001
Top to bottom	2.171	0.886	2.450	0.0143
Side	5.849	1.312	4.459	<0.0001

## Discussion

Our study provided an interesting example to show termite escaping behaviors in the wild, and the results differed from previous laboratory studies. For example, Schwinghammer & Houseman (2006) and Gautam & Henderson (2012) found that termites escaped immediately after disturbance. In the present study, however, the wandering behavior, which usually lasted for several seconds to minutes, was observed before escaping. It is not known why wandering was not observed in laboratory studies examining termite escaping behavior. Under real-world conditions, however, wandering may be an important strategy for termites to probe the environment. Similar behaviors have been observed in some migrating mammals, such as the wildebeest (*Connochaetes taurinus* Burchell), before crossing barriers such as rivers (e.g., see BBC, 2007). The wandering behavior exhibited by termites and many other animals may be the result of convergent evolution for gregarious animals in response to uncertain situations and potential dangers when moving in large groups/herds.

As a eusocial insect, isolated termites usually perform differently from grouped individuals (Traniello, Rosengaus & Savoie, 2002; Hu, Appel & Traniello, 2003). For example, after a vibrational disturbance, grouped *C. formosanus* and *Reticulitermes flavipes* (Kollar) immediately escaped from the disturbed arena through an exit, whereas individual termites exhibited slow, non-directional movements, and only a few individual termites successfully found their way out of the arena (Hu, Appel & Traniello, 2003). In the present study, we found that the escaping speed of individual termites was significantly slower than those that formed escaping flows. Slower escaping speed in individual termites may be due to frequent stops, turns and slow movements in the exposed area, which may be a strategy for detecting potential dangers.

Escaping flows may be triggered by several pioneer termites which have successfully passed through the exposed area of the damaged mud tubes, demonstrating that it is “safe” in the outer environment. Wang et al. (2016) reported that the escaping flow of *C. formosanus* lasted for a long period of time (>40 min) under laboratory conditions. Likewise, long-lasting escaping flows of *O. formosanus* were observed in the present study (Table S2). Given that the damaged mud tube can be repaired relatively quickly, it is unknown why so many termites immediately moved downward instead of waiting for the repair to be completed. One possible explanation is that escaping flow is self-organized by triggering more termites to follow nearby individuals, and therefore the majority of foragers can evacuate from the potential danger in time.

It is important to note that the disturbance was introduced in the lower part of the trunk. The escape of a considerable number of termites from the upper part of the tree should be triggered by signal(s) that can be transmitted long distance. The signal(s) can be chemical or physical. For example, two trail pheromone compounds have been identified in *O. formosanus*, one showed both orientation and recruitment effects during foraging and the other oriented termites to explore new environments (Wen, Ji & Sillam-Dussès, 2014). In addition, alarm pheromones have been identified in many termite species (Leonhardt et al., 2016), although they have not yet been found in *O. formosanus*. Alarm pheromones may initiate termite escaping processes. Another possible mechanism for signal transport in escaping flows is vibrational cues that can travel over long distances. Previous studies have shown that vibrational cues are utilized by termites to explore food quality (e.g., size) and detect potential competitors and predators (Evans et al., 2007; Evans et al., 2009; Oberst et al., 2017). Interestingly, Cristaldo et al. (2015) reported that the alarming behaviors of soldier *Constrictotermes cyphergaster* (Silvestri) were modulated by both pheromonal and vibroacoustic signals. It would be valuable to investigate whether either or both pheromones and vibrational cues play roles in the long-lasting exodus of *O. formosanus*.

Studies on herds, shoals, and flocks indicate that the movement of adjacent individuals in a similar direction and speed is important for stable collective movement (Nagy et al., 2010; Herbert-Read et al., 2011; Herbert-Read et al., 2013; Wang et al., 2016). Likewise, compared to escaping individual, escaping flows resulted in significantly fewer individuals which exhibited upward moving, backward moving, and wandering behaviors. Moreover, no congestion was observed once the escaping flow was formed. In addition, our study showed that the moving speed of termites remained relatively stable after the escaping flow was formed.

Surprisingly, repairing behaviors did not seem to negatively affect evacuation even though it could theoretically present an opportunity for congestion as termites move in opposing directions. A possible explanation may be related to the difference in preferred moving patterns of escaping versus repairing termites. We found that new mud tubes were preferentially repaired from the bottom to the top while escaping termites preferentially moved from the top down. If repairing termites move along the inner tube, away from the trunk while escaping termites move along the trunk of the tree, then there will be no congestion and the “faster-is-slower” effect will be avoided. However, if tubes were preferentially repaired from the top to the bottom, repairing termites would have to move along the tree trunk through the exposed pathway to gain access to the top fracture, thus causing a jam.

Termites are vulnerable to predation when they move across exposed areas. Rather than completely finishing tubes from the start, we observed a “quick and dirty” repair followed by subsequent modifications (e.g., broadening) after the tubes were closed. This strategy shortens the exposure time and may reduce the risk of attack by predators. When ants were present, termites stopped moving and sealed mud tubes, which indicates the repairing and escaping behaviors of termites are flexible and modified by biotic factors.

Traditionally, baits and liquid pesticides have been widely used for termite control (Rust & Su, 2012). Both methods are dependent on the horizontal transfer of slow-acting toxicants among nest-mates of termites (Rust & Su, 2012; Evans & Iqbal, 2015). However, baiting is not as successful against higher termites (Evans & Iqbal, 2015). Soil treatment using pesticides is also unpractical to apply in large forest areas to reduce *O. formosanus* populations. Our study might provide new insight for the control of *O. formosanus*. Liquid termiticides can be sprayed on damaged mud tubes where termites would come in contact and carry the toxicant as they escape and repair the damaged area. In addition, essential oils extracted from pepper-rosmarin (*Lippia sidoides* Cham.) and patchouli (*Pogostemon cablin* [Blanco] Benth.) reduced the collective interactions and walking abilities of various termites (Bacci et al., 2015; Santos et al., 2017). Adding these essential oils on the broken mud tubes may interfere with the escaping behaviors of *O. formosanus*. Future investigation of these novel methods would be valuable.

##  Supplemental Information

10.7717/peerj.4513/supp-1 Table S1 General information about each termite colony/subcolony group and disturbance test

10.7717/peerj.4513/supp-2 Table S2 Time point for termite repairing and escaping behaviors for each disturbance test

10.7717/peerj.4513/supp-3 Table S3 Summary of generalized linear mixed-effect models comparing the number of termite individuals that performed downward escaping with the number performing other behaviors after the mud tube had been damaged

10.7717/peerj.4513/supp-4 Table S4 Summary of post-hoc comparisons (Tukey’s Honestly Significant Difference tests) among the number of termites that exhibited downward moving, repairing, turning/backward moving, upward moving, and wandering at each time interval

10.7717/peerj.4513/supp-5 Table S5 Summary of post-hoc comparisons (Tukey’s Honestly Significant Difference tests) among speed of termites in the individual escaping phase and three periods of escaping flow phase

10.7717/peerj.4513/supp-6 Table S6 Summary of generalized linear mixed-effect models comparing the repairing area constructed from bottom to top with other directions of the termite after the mud tube had been damaged

10.7717/peerj.4513/supp-7 Table S7 Summary of post-hoc comparisons (Tukey’s Honestly Significant Difference tests) among areas that were repaired from bottom to top, top to bottom, and side at each time interval

10.7717/peerj.4513/supp-8 Data S1 Escaping speed of termites in the individual escaping phase and three periods of escaping flow phase (5–6 min after the escaping flow was formed, in the middle of the escaping flow duration, and 5–6 min before the escaping flow ended)

10.7717/peerj.4513/supp-9 Data S2 Length and areas of mud tubes that were repaired from bottom to top, top to bottom, and side at each time interval

10.7717/peerj.4513/supp-10 Data S3 Number of termites that exhibited downward moving, repairing, turning/backward moving, upward moving, and wandering at each time interval

10.7717/peerj.4513/supp-11 Data S4 Number of termites that exhibited downward moving, repairing, turning/backward moving, upward moving and wandering when the predators (ants) were presented
