# Supplementary material for: Escaping and repairing behaviors of the termite Odontotermes formosanus (Blattodea: Termitidae) in response to disturbance
Source: PeerJ. 2018 Mar 16;6:e4513. doi: 10.7717/peerj.4513 (PMC5858535; doi:10.7717/peerj.4513)
Supplement: Table S1 [file peerj-06-4513-s001.docx]

Table S1. General information about each termite colony/subcolony group and disturbance test. C: colony, TS: tree status, D: diameter (measured at the height where the mud tube was damaged), T: temperature, ST: starting time, W: weather.

| **C** | **Host plant and location information** | | | | **First disturbance test** | | | | **Second disturbance test** | | | |
| --- | --- | --- | --- | --- | --- | --- | --- | --- | --- | --- | --- | --- |
|  | **Species** | **TS** | **D (cm)** | **Location** | **Date**  **(dd/mm/yyyy)** | **ST** | **W** | **T (°C)** | **Date**  **(dd/mm/yyyy)** | **ST** | **W** | **T (°C)** |
| 1 | *Eucalyptus* sp. | dead | 14.5 | 23°9′19′′N,  113°21′30′′E | 05/09/2016 | 15:55 | sunny | 30 | 12/09/2016 | 12:40 | sunny/  cloudy | 30 |
| 2 | *Eucalyptus* sp. | live | 31.9 | 23°9′17′′N,  113°21′28′′E | 06/09/2016 | 15:00 | overcast | 29 |  |  |  |  |
| 3 | *Eucalyptus* sp. | live | 33.0 | 23°9′18′′N,  113°21′28′′E | 11/09/2016 | 11:00 | cloudy/  sunny | 28 | 13/09/2016 | 9:20 | sunny | 29 |
| 4 | *Acacia confusa* | live | 20.8 | 23°9′19′′N,  113°21′29′′E | 12/09/2016 | 13:45 | sunny | 31 | 14/09/2016 | 9:05 | sunny | 28 |
| 5 | *Eucalyptus* sp. | live | 30.9 | 23°9′21′′N,  113°21′29′′E | 12/09/2016 | 17:15 | overcast | 29 | 14/09/2016 | 9:55 | sunny | 28 |
| 6 | *Eucalyptus* sp. | dead | 20.2 | 23°9′18′′N,  113°21′31′′E | 18/09/2016 | 16:40 | sunny | 28 | 21/09/2016 | 8:30 | sunny | 26 |
| 7 | *Eucalyptus* sp. | live | 17.1 | 23°9′9′′N,  113°21′50′′E | 13/09/2016 | 16:35 | sunny | 31 | 15/09/2016 | 10:30 | sunny | 33 |
| 8 | *Eucalyptus* sp. | live | 29.1 | 23°9′10′′N,  113°21′48′′E | 14/09/2016 | 14:50 | sunny | 31 | 16/09/2016 | 14:05 | sunny | 34 |
| 9 | *Eucalyptus* sp. | dead | 13.4 | 23°9′9′′N,  113°21′51′′E | 16/09/2016 | 16:05 | sunny | 32 | 18/09/2016 | 11:10 | sunny | 29 |
| 10 | *Schima superba* | live | 21.5 | 23°9′13′′N,  113°21′41′′E | 17/09/2016 | 9:50 | sunny | 30 | 20/09/2016 | 9:20 | overcast | 27 |
| 11 | *Eucalyptus* sp. | live | 31.9 | 23°9′19′′N,  113°21′30′′E | 18/09/2016 | 14:45 | sunny | 30 | 21/09/2016 | 10:15 | sunny | 27 |
| 12 | *Eucalyptus* sp. | dead | 23.2 | 23°9′18′′N,  113°21′34′′E | 19/09/2016 | 16:00 | sunny | 30 | 25/09/2016 | 15:40 | sunny | 30 |
| 13 | *Dolichandrone caudafelina* | live | 50.8 | 23°9′29′′N,  113°21′1′′E | 24/09/2016 | 9:30 | cloudy | 30 | 26/09/2016 | 14:20 | sunny | 37 |
| 14 | *Cinnamomum camphora* | live | 45.3 | 23°9′24′′N,  113°21′2′′E | 24/09/2016 | 11:20 | cloudy | 29 | 26/09/2016 | 10:50 | cloudy/  sunny | 35 |
| 15 | *Cinnamomum camphora* | live | 60.1 | 23°9′23′′N,  113°21′2′′E | 24/09/2016 | 13:05 | cloudy | 30 | 26/09/2016 | 12:45 | cloudy/  sunny | 31 |
| 16 | *Eucalyptus* sp. | live | 35.1 | 23°9′19′′N,  113°21′28′′E | 17/10/2016 | 10:20 | cloudy | 28 |  |  |  |  |
| 17 | *Eucalyptus* sp. | live | 42.5 | 23°9′14′′N,  113°21′28′′E | 17/10/2016 | 12:10 | cloudy | 29 |  |  |  |  |
| 18 | *Eucalyptus* sp. | dead | 23.1 | 23°9′18′′N,  113°21′24′′E | 23/10/2016 | 10:55 | cloudy | 28 | 27/10/2016 | 10:00 | sunny | 29 |
| 19 | *Tectona grandis* | live | 56.5 | 23°9′22′′N,  113°21′2′′E | 24/10/2016 | 13:00 | cloudy | 29 | 29/10/2016 | 9:30 | cloudy | 27 |
| 20 | *Eucalyptus exserta* | live | 44.0 | 23°9′22′′N,  113°21′3′′E | 25/10/2016 | 16:00 | cloudy | 30 | 31/10/2016 | 9:50 | cloudy | 25 |
| 21 | *Tectona grandis* | live | 91.4 | 23°9′20′′N,  113°21′5′′E | 26/10/2016 | 15:30 | sunny | 32 | 01/11 /2016 | 10:50 | cloudy | 24 |
| 22 | *Eucalyptus exserta* | live | 52.2 | 23°9′28′′N,  113°20′56′′E | 28/10/2016 | 10:45 | sunny | 31 | 04/11/2016 | 9:30 | sunny | 21 |
