# Supplementary material for: Escaping and repairing behaviors of the termite Odontotermes formosanus (Blattodea: Termitidae) in response to disturbance
Source: PeerJ. 2018 Mar 16;6:e4513. doi: 10.7717/peerj.4513 (PMC5858535; doi:10.7717/peerj.4513)
Supplement: Table S2 [file peerj-06-4513-s002.docx]

**Table S2:** Time point (min: sec) for termite repairing and escaping behaviors for each disturbance test.

| **Colony group** | **Video No.** | **Type** | **Repairing behavior** | | **Escaping behavior** | | | |
| --- | --- | --- | --- | --- | --- | --- | --- | --- |
|  |  |  | **Repairing starting** | **Mud-tube closed** | **Wandering starting** | **Individual escaping starting** | **Escaping flow starting** | **Escaping flow ending** |
| 1 | 1 | Ⅰ | 00:51 | 36:26 | 00:00 | 00:09 | 08:21 | NA^a^ |
|  | 2 | Ⅰ | 00:39 | 32:14 | 00:01 | 07:10 | 08:20 | NA |
| 2 | 3 | Ⅰ | 05:01 | 52:06 | 00:04 | 04:15 | 06:57 | NA |
|  | ̵̶̶ ^b^ |  |  |  |  |  |  |  |
| 3 | 4 | Ⅰ | 00:06 | 39:36 | 00:00 | 07:24 | 09:13 | NA |
|  | 5 | Ⅱ | 02:20 | ̵̶̶ ^c^ | 00:14 | 00:21 | 02:22 | 32:41 |
| 4 | 6 | Ⅲ | 00:13 | 92:07 | 00:06 | ̵̶̶ ^d^ | ̵̶̶ ^e^ |  |
|  | 7 | Ⅳ | 01:35 | ̵̶̶ ^c^ | 00:18 | 07:36 | 07:51 | 09:46 |
| 5 | 8 | Ⅰ | 01:20 | 49:17 | 00:01 | 00:34 | 14:32 | NA |
|  | 9 | Ⅰ | 01:42 | 61:35 | 02:11 | 21:22 | 21:42 | NA |
| 6 | 10 | Ⅰ | 00:10 | 35:15 | 00:00 | 00:11 | 00:57 | NA |
|  | 11 | Ⅰ | 00:13 | 46:47 | 00:00 | 00:07 | 00:07 | NA |
| 7 | 12 | Ⅰ | 00:05 | 29:09 | 00:00 | 00:04 | 00:17 | NA |
|  | 13 | Ⅰ | 00:13 | 34:47 | 00:00 | 00:04 | 00:04 | NA |
| 8 | 14 | Ⅰ | 01:00 | 57:56 | 00:03 | 00:21 | 01:13 | 37:00 |
|  | 15 | Ⅲ | 01:07 | 97:53 | 00:04 | 14:43 | ̵̶̶ ^e^ |  |
| 9 | 16 | Ⅰ | 04:29 | 105:00 | 00:00 | 06:29 | 08:16 | 17:30 |
|  | 17 | Ⅰ | 00:48 | 54:21 | 00:01 | 01:26 | 04:13 | 11:41 |
| 10 | 18 | Ⅰ | 00:12 | 39:07 | 00:00 | 00:45 | 02:01 | NA |
|  | 19 | Ⅰ | 01:17 | 47:44 | 00:00 | 00:36 | 00:43 | NA |
| 11 | 20 | Ⅰ | 00:14 | 48:50 | 00:00 | 00:03 | 00:03 | NA |
|  | 21 | Ⅰ | 00:36 | 79:23 | 00:05 | 00:26 | 02:54 | 15:19 |
| 12 | 22 | Ⅰ | 00:17 | 29:28 | 00:00 | 00:08 | 00:11 | NA |
|  | 23 | Ⅰ | 00:18 | 29:21 | 00:00 | 00:07 | 00:09 | NA |
| 13 | 24 | Ⅰ | 02:02 | 53:45 | 00:07 | 00:14 | 02:37 | 41:59 |
|  | 25 | Ⅲ | 00:28 | 55:50 | 00:20 | 00:33 | ̵̶̶ ^e^ |  |
| 14 | 26 | Ⅰ | 00:12 | 23:49 | 00:00 | 00:03 | 00:03 | NA |
|  | 27 | Ⅰ | 00:45 | 22:14 | 00:00 | 00:12 | 00:52 | NA |
| 15 | 28 | Ⅰ | 00:22 | 16:11 | 00:00 | 00:18 | 00:18 | NA |
|  | 29 | Ⅰ | 01:22 | 27:15 | 00:00 | 00:08 | 05:39 | NA |
| 16 | 30 | Ⅰ | 00:07 | 31:59 | 00:00 | 00:08 | 00:18 | NA |
|  | ̵̶̶ ^b^ |  |  |  |  |  |  |  |
| 17 | 31 | Ⅰ | 02:30 | 58:43 | 00:00 | 00:12 | 02:50 | NA |
|  | ̵̶̶ ^b^ |  |  |  |  |  |  |  |
| 18 | 32 | Ⅰ | 01:03 | 38:59 | 00:00 | 02:08 | 02:40 | NA |
|  | 33 | Ⅱ | 00:33 | ̵̶̶ ^c^ | 00:00 | 05:40 | 06:27 | 52:00 |
| 19 | 34 | Ⅱ | 00:28 | 19:59 | 00:00 | 00:14 | 00:24 | NA |
|  | 35 | Ⅱ | 02:04 | ̵̶̶ ^c^ | 00:00 | 00:15 | 05:34 | 15:04 |
| 20 | 36 | Ⅱ | 03:17 | ̵̶̶ ^c^ | 00:06 | 00:43 | ̵̶̶ ^e^ |  |
|  | 37 | Ⅱ | 03:04 | ̵̶̶ ^c^ | 00:22 | 01:38 | 14:50 | 32:57 |
| 21 | 38 | Ⅰ | 00:19 | 27:01 | 00:01 | 00:08 | 00:08 | NA |
|  | 39 | Ⅰ | 00:33 | 41:06 | 00:00 | 00:05 | 00:07 | NA |
| 22 | 40 | Ⅰ | 00:34 | 27:34 | 00:04 | 00:40 | 01:54 | NA |
|  | 41 | Ⅰ | 02:01 | 106:59 | 00:03 | 00:11 | 00:51 | NA |

^a^ Escaping flow did not stop when the mud tube was closed.

^b^ Mud tubes were damaged by rainstorm so that we did not conduct the second disturbance test.

^c^ Mud tubes were not closed within the 2-h period.

^d^ Individual escaping was not observed within the 2-h period.

^e^ Escaping flows were not observed within the 2-h period.
