# Supplementary material for: Escaping and repairing behaviors of the termite Odontotermes formosanus (Blattodea: Termitidae) in response to disturbance
Source: PeerJ. 2018 Mar 16;6:e4513. doi: 10.7717/peerj.4513 (PMC5858535; doi:10.7717/peerj.4513)
Supplement: Table S3 [file peerj-06-4513-s003.docx]

Table S3: Summary of generalized linear mixed-effect models comparing the number of termite (*Odontotermes formosanus*) individuals that performed downward escaping with the number performing other behaviors after the mud tube had been damaged. DM: downward moving, RP: repairing, TB: turning/backward moving, UM: upward moving, W: wandering. The number following “time” indicates the time (minute) after tubes were damaged. “:” indicates the interaction between behavior and time

a) Damaged mud tubes were closed within 20 minute

|  | **Estimate** | **SE** | **Z value** | ***P*** |
| --- | --- | --- | --- | --- |
| (Intercept) | 2.6693 | 0.2045 | 13.055 | <.0001 |
| RP | -3.1034 | 0.5454 | -5.69 | <.0001 |
| TB | -1.0554 | 0.2883 | -3.66 | 0.0003 |
| UM | -3.8379 | 0.7642 | -5.022 | <.0001 |
| W | -0.7452 | 0.2951 | -2.525 | 0.0116 |
| time2 | 0.8845 | 0.122 | 7.252 | <.0001 |
| time4 | 1.2148 | 0.1169 | 10.396 | <.0001 |
| time6 | 1.3627 | 0.115 | 11.849 | <.0001 |
| time8 | 1.221 | 0.1168 | 10.456 | <.0001 |
| time10 | 1.1536 | 0.1177 | 9.802 | <.0001 |
| time12 | 0.9679 | 0.1205 | 8.03 | <.0001 |
| time14 | 1.2118 | 0.1256 | 9.646 | <.0001 |
| time16 | 1.0095 | 0.13 | 7.767 | <.0001 |
| time18 | 0.7995 | 0.1496 | 5.344 | <.0001 |
| time20 | 0.7455 | 0.2477 | 3.009 | 0.0026 |
| time22 | 1.1348 | 0.2145 | 5.29 | <.0001 |
| RP:time2 | 1.9646 | 0.5293 | 3.712 | 0.0002 |
| TB:time2 | -1.0604 | 0.2931 | -3.618 | 0.0003 |
| UM:time2 | 1.5584 | 0.748 | 2.084 | 0.0372 |
| W:time2 | -2.0518 | 0.3309 | -6.201 | <.0001 |
| RP:time4 | 1.8775 | 0.5252 | 3.575 | 0.0004 |
| TB:time4 | -1.0113 | 0.2693 | -3.756 | 0.0002 |
| UM:time4 | 1.1371 | 0.7499 | 1.516 | 0.1294 |
| W:time4 | -2.824 | 0.3852 | -7.331 | <.0001 |
| RP:time6 | 1.8166 | 0.5239 | 3.468 | 0.0005 |
| TB:time6 | -1.1857 | 0.2698 | -4.395 | <.0001 |
| UM:time6 | 1.1635 | 0.7445 | 1.563 | 0.1181 |
| W:time6 | -3.0897 | 0.4025 | -7.677 | <.0001 |
| RP:time8 | 1.9158 | 0.5247 | 3.651 | 0.0003 |
| TB:time8 | -1.3593 | 0.2885 | -4.711 | <.0001 |
| UM:time8 | 1.9151 | 0.7324 | 2.615 | 0.0089 |
| W:time8 | -2.8302 | 0.3852 | -7.348 | <.0001 |
| RP:time10 | 1.9612 | 0.5251 | 3.735 | 0.0002 |
| TB:time10 | -1.4522 | 0.2999 | -4.842 | <.0001 |
| UM:time10 | 2.3282 | 0.7282 | 3.197 | 0.0014 |
| W:time10 | -3.3503 | 0.4882 | -6.863 | <.0001 |
| RP:time12 | 2.2622 | 0.5246 | 4.312 | <.0001 |
| TB:time12 | -1.4062 | 0.3117 | -4.511 | <.0001 |
| UM:time12 | 2.8505 | 0.7256 | 3.928 | <.0001 |
| W:time12 | -3.1647 | 0.4889 | -6.473 | <.0001 |
| RP:time14 | 1.78 | 0.5346 | 3.33 | 0.0009 |
| TB:time14 | -1.2245 | 0.3239 | -3.781 | 0.0002 |
| UM:time14 | 2.4542 | 0.7374 | 3.328 | 0.0009 |
| W:time14 | -3.6708 | 0.613 | -5.988 | <.0001 |
| RP:time16 | 2.0364 | 0.5347 | 3.809 | 0.0001 |
| TB:time16 | -0.9169 | 0.3169 | -2.893 | 0.0038 |
| UM:time16 | 3.0035 | 0.7333 | 4.096 | <.0001 |
| W:time16 | -3.1806 | 0.5411 | -5.878 | <.0001 |
| RP:time18 | 2.261 | 0.5456 | 4.144 | <.0001 |
| TB:time18 | -1.1084 | 0.3958 | -2.801 | 0.0051 |
| UM:time18 | 3.3791 | 0.738 | 4.579 | <.0001 |
| W:time18 | -4.1159 | 1.0277 | -4.005 | <.0001 |
| RP:time20 | 2.2653 | 0.6337 | 3.575 | 0.0004 |
| TB:time20 | -1.3903 | 0.775 | -1.794 | 0.0728 |
| UM:time20 | 3.2676 | 0.8032 | 4.068 | <.0001 |
| W:time20 | -18.292 | 1786.826 | -0.01 | 0.9918 |
| RP:time22 | 2.0993 | 0.6079 | 3.453 | 0.0006 |
| TB:time22 | -2.4724 | 1.0428 | -2.371 | 0.0177 |
| UM:time22 | 3.235 | 0.78 | 4.148 | <.0001 |
| W:time22 | -18.6627 | 1770.272 | -0.011 | 0.9916 |
| UM:time22 | 3.235 | 0.78 | 4.148 | <.0001 |
| W:time22 | -18.6627 | 1770.272 | -0.011 | 0.9916 |

b) Damaged mud tubes were closed between 20 and 40 minute

|  | **Estimate** | **SE** | **Z value** | ***P*** |
| --- | --- | --- | --- | --- |
| (Intercept) | 2.38715 | 0.13227 | 18.047 | <.0001 |
| RP | -2.11572 | 0.25506 | -8.295 | <.0001 |
| TB | -1.35797 | 0.20322 | -6.682 | <.0001 |
| UM | -2.35877 | 0.30784 | -7.662 | <.0001 |
| W | -0.76319 | 0.20235 | -3.772 | 0.0002 |
| time2 | 0.21189 | 0.10075 | 2.103 | 0.0355 |
| time4 | 0.51871 | 0.09461 | 5.482 | <.0001 |
| time6 | 0.64723 | 0.09246 | 7 | <.0001 |
| time8 | 0.93641 | 0.08838 | 10.595 | <.0001 |
| time10 | 0.68759 | 0.09183 | 7.488 | <.0001 |
| time12 | 0.58969 | 0.0934 | 6.314 | <.0001 |
| time14 | 0.73447 | 0.09112 | 8.06 | <.0001 |
| time16 | 0.74781 | 0.09093 | 8.224 | <.0001 |
| time18 | 0.99622 | 0.08766 | 11.365 | <.0001 |
| time20 | 1.02483 | 0.08732 | 11.736 | <.0001 |
| time22 | 0.89131 | 0.08895 | 10.02 | <.0001 |
| time24 | 0.93862 | 0.08835 | 10.624 | <.0001 |
| time26 | 0.85104 | 0.09251 | 9.199 | <.0001 |
| time28 | 1.04628 | 0.09225 | 11.342 | <.0001 |
| time30 | 1.01023 | 0.09591 | 10.533 | <.0001 |
| time32 | 0.78821 | 0.10588 | 7.445 | <.0001 |
| time34 | 1.01174 | 0.14747 | 6.861 | <.0001 |
| time36 | 0.8298 | 0.26746 | 3.103 | 0.0019 |
| time38 | 1.38924 | 0.21165 | 6.564 | <.0001 |
| time40 | 1.61264 | 0.19404 | 8.311 | <.0001 |
| RP:time2 | 1.25876 | 0.26745 | 4.706 | <.0001 |
| TB:time2 | -0.10752 | 0.22832 | -0.471 | 0.6377 |
| UM:time2 | 0.95801 | 0.2874 | 3.333 | 0.0009 |
| W:time2 | -1.20376 | 0.22592 | -5.328 | <.0001 |
| RP:time4 | 1.23094 | 0.25988 | 4.737 | <.0001 |
| TB:time4 | -0.02217 | 0.2109 | -0.105 | 0.9163 |
| UM:time4 | 0.2796 | 0.29853 | 0.937 | 0.34893 |
| W:time4 | -2.01118 | 0.26309 | -7.644 | <.0001 |
| RP:time6 | 1.29194 | 0.2562 | 5.043 | <.0001 |
| TB:time6 | -0.58365 | 0.22662 | -2.575 | 0.01 |
| UM:time6 | 0.5227 | 0.28458 | 1.837 | 0.0662 |
| W:time6 | -1.99993 | 0.24978 | -8.007 | <.0001 |
| RP:time8 | 1.19216 | 0.25231 | 4.725 | <.0001 |
| TB:time8 | -0.27807 | 0.2033 | -1.368 | 0.1713 |
| UM:time8 | 0.16208 | 0.28553 | 0.568 | 0.5702 |
| W:time8 | -2.59155 | 0.27706 | -9.354 | <.0001 |
| RP:time10 | 1.36066 | 0.25452 | 5.346 | <.0001 |
| TB:time10 | -0.32089 | 0.21406 | -1.499 | 0.1338 |
| UM:time10 | 1.03698 | 0.27123 | 3.823 | 0.0001 |
| W:time10 | -1.95706 | 0.24262 | -8.066 | <.0001 |
| RP:time12 | 1.55087 | 0.25398 | 6.106 | <.0001 |
| TB:time12 | -0.23853 | 0.21529 | -1.108 | 0.2678 |
| UM:time12 | 1.2647 | 0.26955 | 4.692 | <.0001 |
| W:time12 | -1.98705 | 0.25401 | -7.823 | <.0001 |
| RP:time14 | 1.496 | 0.25215 | 5.933 | <.0001 |
| TB:time14 | -0.16012 | 0.20694 | -0.774 | 0.4391 |
| UM:time14 | 0.89676 | 0.27276 | 3.288 | 0.001 |
| W:time14 | -3.14325 | 0.37729 | -8.331 | <.0001 |
| RP:time16 | 1.54012 | 0.25148 | 6.124 | <.0001 |
| TB:time16 | -0.32123 | 0.21159 | -1.518 | 0.1289 |
| UM:time16 | 1.03454 | 0.2699 | 3.833 | 0.0001 |
| W:time16 | -3.1564 | 0.37723 | -8.367 | <.0001 |
| RP:time18 | 1.22344 | 0.25103 | 4.874 | <.0001 |
| TB:time18 | -0.70946 | 0.21528 | -3.296 | 0.0009 |
| UM:time18 | 0.69819 | 0.2704 | 2.582 | 0.0098 |
| W:time18 | -3.08659 | 0.32896 | -9.383 | <.0001 |
| RP:time20 | 1.23217 | 0.25052 | 4.918 | <.0001 |
| TB:time20 | -0.67346 | 0.21272 | -3.166 | 0.0015 |
| UM:time20 | 0.83821 | 0.26736 | 3.135 | 0.0017 |
| W:time20 | -3.72136 | 0.42738 | -8.707 | <.0001 |
| RP:time22 | 1.39663 | 0.25077 | 5.569 | <.0001 |
| TB:time22 | -0.40849 | 0.20886 | -1.956 | 0.0504 |
| UM:time22 | 1.11606 | 0.26574 | 4.2 | <.0001 |
| W:time22 | -3.5878 | 0.42775 | -8.388 | <.0001 |
| RP:time24 | 1.36443 | 0.25041 | 5.449 | <.0001 |
| TB:time24 | -0.5121 | 0.21049 | -2.433 | 0.0149 |
| UM:time24 | 1.23344 | 0.26341 | 4.683 | <.0001 |
| W:time24 | -3.48099 | 0.39925 | -8.719 | <.0001 |
| RP:time26 | 1.45871 | 0.25344 | 5.756 | <.0001 |
| TB:time26 | -0.38786 | 0.21841 | -1.776 | 0.0757 |
| UM:time26 | 1.50276 | 0.26523 | 5.666 | <.0001 |
| W:time26 | -3.80516 | 0.51591 | -7.376 | <.0001 |
| RP:time28 | 1.34689 | 0.25563 | 5.269 | <.0001 |
| TB:time28 | -0.42219 | 0.22249 | -1.898 | 0.0577 |
| UM:time28 | 1.22735 | 0.27219 | 4.509 | <.0001 |
| W:time28 | -4.55141 | 0.71635 | -6.354 | <.0001 |
| RP:time30 | 1.43107 | 0.25875 | 5.531 | <.0001 |
| TB:time30 | -0.53237 | 0.23979 | -2.22 | 0.0264 |
| UM:time30 | 1.56065 | 0.27119 | 5.755 | <.0001 |
| W:time30 | -4.31417 | 0.71735 | -6.014 | <.0001 |
| RP:time32 | 1.7169 | 0.26672 | 6.437 | <.0001 |
| TB:time32 | -1.10768 | 0.3247 | -3.411 | 0.0006 |
| UM:time32 | 1.71695 | 0.27927 | 6.148 | <.0001 |
| W:time32 | -3.18302 | 0.52143 | -6.104 | <.0001 |
| RP:time34 | 1.32246 | 0.33521 | 3.945 | <.0001 |
| TB:time34 | -1.03826 | 0.46352 | -2.24 | 0.0251 |
| UM:time34 | 0.72287 | 0.41474 | 1.743 | 0.0813 |
| W:time34 | -17.4788 | 57.34828 | -0.305 | 0.7605 |
| RP:time36 | 1.14526 | 0.50106 | 2.286 | 0.0222 |
| TB:time36 | -1.95804 | 1.04808 | -1.868 | 0.0617 |
| UM:time36 | 2.36641 | 0.40854 | 5.792 | <.0001 |
| W:time36 | -16.5482 | 68.84636 | -0.24 | 0.81 |
| RP:time38 | 0.45197 | 0.49213 | 0.918 | 0.3584 |
| TB:time38 | -1.41935 | 0.63917 | -2.221 | 0.0263 |
| UM:time38 | 1.54122 | 0.38766 | 3.976 | <.0001 |
| W:time38 | -17.2405 | 128.1646 | -0.135 | 0.8929 |
| RP:time40 | 0.47997 | 0.45095 | 1.064 | 0.2871 |
| TB:time40 | -2.04867 | 0.75339 | -2.719 | 0.0065 |
| UM:time40 | 0.89032 | 0.40777 | 2.183 | 0.029 |
| W:time40 | -17.3827 | 97.09811 | -0.179 | 0.8579 |

c) Damaged mud tubes were closed beyond 40 minute

|  | **Estimate** | **SE** | **Z value** | ***P*** |
| --- | --- | --- | --- | --- |
| (Intercept) | 1.186 | 0.3153 | 3.762 | 0.0002 |
| RP | -2.35 | 0.6275 | -3.745 | 0.0002 |
| TB | -0.7451 | 0.3197 | -2.331 | 0.0198 |
| UM | -3.391 | 0.7535 | -4.501 | <.0001 |
| W | -0.7533 | 0.4223 | -1.784 | 0.0745 |
| time2 | 0.1615 | 0.215 | 0.751 | 0.4526 |
| time4 | 0.8652 | 0.1884 | 4.592 | <.0001 |
| time6 | 1.115 | 0.1821 | 6.124 | <.0001 |
| time8 | 1.107 | 0.1823 | 6.072 | <.0001 |
| time10 | 1.194 | 0.1804 | 6.618 | <.0001 |
| time12 | 0.8111 | 0.19 | 4.27 | <.0001 |
| time14 | 0.9653 | 0.1857 | 5.197 | <.0001 |
| time16 | 0.3003 | 0.2085 | 1.44 | 0.1498 |
| time18 | 0.4058 | 0.204 | 1.989 | 0.0467 |
| time20 | 0.5598 | 0.1981 | 2.825 | 0.0047 |
| time22 | 0.4858 | 0.2009 | 2.418 | 0.0155 |
| time24 | -0.1051 | 0.2296 | -0.458 | 0.6472 |
| time26 | -0.1333 | 0.2314 | -0.576 | 0.5645 |
| time28 | -0.6925 | 0.2737 | -2.53 | 0.0114 |
| time30 | -0.5108 | 0.2581 | -1.979 | 0.0478 |
| time32 | 0.3889 | 0.2047 | 1.899 | 0.0575 |
| time34 | 0.5309 | 0.1992 | 2.665 | 0.0076 |
| time36 | -0.2229 | 0.2371 | -0.94 | 0.3471 |
| time38 | -0.3563 | 0.2463 | -1.447 | 0.148 |
| time40 | -0.6931 | 0.2738 | -2.532 | 0.0113 |
| time42 | -0.3565 | 0.2463 | -1.447 | 0.1477 |
| time44 | -0.5078 | 0.2788 | -1.821 | 0.0685 |
| time46 | 0.2851 | 0.2213 | 1.289 | 0.1975 |
| time48 | -0.2387 | 0.2744 | -0.87 | 0.3844 |
| time50 | -0.04282 | 0.3121 | -0.137 | 0.8908 |
| time52 | 0.1517 | 0.2912 | 0.521 | 0.6023 |
| time54 | 0.0045 | 0.3211 | 0.014 | 0.9886 |
| RP:time2 | 1.38 | 0.6718 | 2.054 | 0.0399 |
| TB:time2 | 0.3247 | 0.3835 | 0.846 | 0.3972 |
| UM:time2 | 0.9366 | 0.8455 | 1.108 | 0.2679 |
| W:time2 | 0.266 | 0.3964 | 0.671 | 0.502 |
| RP:time4 | 1.333 | 0.6373 | 2.091 | 0.0365 |
| TB:time4 | -1.152 | 0.4256 | -2.707 | 0.0067 |
| UM:time4 | 1.214 | 0.7743 | 1.567 | 0.117 |
| W:time4 | -0.6287 | 0.3945 | -1.594 | 0.1109 |
| RP:time6 | 1.398 | 0.6275 | 2.227 | 0.0259 |
| TB:time6 | -0.7091 | 0.3704 | -1.914 | 0.0555 |
| UM:time6 | 0.2704 | 0.8124 | 0.333 | 0.7392 |
| W:time6 | -1.426 | 0.438 | -3.255 | 0.0011 |
| RP:time8 | 1.556 | 0.6246 | 2.491 | 0.0127 |
| TB:time8 | -0.4778 | 0.3591 | -1.33 | 0.1833 |
| UM:time8 | 0.5973 | 0.7911 | 0.755 | 0.4502 |
| W:time8 | -1.25 | 0.4217 | -2.965 | 0.003 |
| RP:time10 | 1.579 | 0.6221 | 2.538 | 0.0111 |
| TB:time10 | -0.5988 | 0.3598 | -1.664 | 0.096 |
| UM:time10 | 0.1916 | 0.812 | 0.236 | 0.8134 |
| W:time10 | -1.263 | 0.4142 | -3.049 | 0.0022 |
| RP:time12 | 2.061 | 0.6234 | 3.306 | 0.0009 |
| TB:time12 | -0.405 | 0.3744 | -1.082 | 0.279 |
| UM:time12 | 1.268 | 0.7747 | 1.636 | 0.1017 |
| W:time12 | -1.44 | 0.4786 | -3.008 | 0.0026 |
| RP:time14 | 1.787 | 0.624 | 2.864 | 0.004 |
| TB:time14 | -0.5183 | 0.37 | -1.401 | 0.1612 |
| UM:time14 | 0.826 | 0.7871 | 1.049 | 0.2939 |
| W:time14 | -1.594 | 0.4769 | -3.342 | 0.0008 |
| RP:time16 | 2.533 | 0.6298 | 4.022 | <.0001 |
| TB:time16 | 0.0188 | 0.389 | 0.049 | 0.9612 |
| UM:time16 | 1.645 | 0.7852 | 2.095 | 0.0361 |
| W:time16 | -1.062 | 0.5044 | -2.106 | 0.0352 |
| RP:time18 | 2.408 | 0.6287 | 3.83 | 0.0001 |
| TB:time18 | 0.0005 | 0.3817 | 0.001 | 0.9990 |
| UM:time18 | 1.098 | 0.809 | 1.357 | 0.1747 |
| W:time18 | -1.322 | 0.5259 | -2.514 | 0.0119 |
| RP:time20 | 2.349 | 0.6253 | 3.757 | 0.0001 |
| TB:time20 | -0.336 | 0.3894 | -0.863 | 0.3882 |
| UM:time20 | 1.637 | 0.7723 | 2.12 | 0.034 |
| W:time20 | -1.322 | 0.5002 | -2.643 | 0.0082 |
| RP:time22 | 2.328 | 0.6277 | 3.709 | 0.0002 |
| TB:time22 | -0.1667 | 0.385 | -0.433 | 0.6650 |
| UM:time22 | 0.6124 | 0.842 | 0.727 | 0.4670 |
| W:time22 | -1.402 | 0.5247 | -2.672 | 0.0075 |
| RP:time24 | 3.032 | 0.6358 | 4.77 | <.0001 |
| TB:time24 | -0.0278 | 0.4319 | -0.064 | 0.9485 |
| UM:time24 | 1.203 | 0.8493 | 1.417 | 0.1565 |
| W:time24 | -0.5235 | 0.4957 | -1.056 | 0.2909 |
| RP:time26 | 3.006 | 0.6372 | 4.717 | <.0001 |
| TB:time26 | -0.336 | 0.4646 | -0.723 | 0.4695 |
| UM:time26 | 1.925 | 0.7991 | 2.409 | 0.0160 |
| W:time26 | -0.9652 | 0.5674 | -1.701 | 0.0889 |
| RP:time28 | 3.655 | 0.6525 | 5.601 | <.0001 |
| TB:time28 | 0.3184 | 0.4777 | 0.667 | 0.5049 |
| UM:time28 | 1.791 | 0.8623 | 2.077 | 0.0378 |
| W:time28 | -0.2238 | 0.5566 | -0.402 | 0.6877 |
| RP:time30 | 3.473 | 0.6461 | 5.375 | <.0001 |
| TB:time30 | 0.8743 | 0.4153 | 2.105 | 0.0352 |
| UM:time30 | 2.215 | 0.8119 | 2.728 | 0.0063 |
| W:time30 | 0.0001 | 0.4956 | 0 | 0.9997 |
| RP:time32 | 2.656 | 0.6256 | 4.246 | <.0001 |
| TB:time32 | -0.165 | 0.3928 | -0.42 | 0.6744 |
| UM:time32 | 1.557 | 0.7842 | 1.985 | 0.0471 |
| W:time32 | -1.71 | 0.6006 | -2.848 | 0.0044 |
| RP:time34 | 2.432 | 0.6249 | 3.891 | <.0001 |
| TB:time34 | -0.5301 | 0.4056 | -1.307 | 0.1912 |
| UM:time34 | 1.484 | 0.7797 | 1.903 | 0.057 |
| W:time34 | -1.853 | 0.5987 | -3.094 | 0.0019 |
| RP:time36 | 3.132 | 0.6388 | 4.904 | <.0001 |
| TB:time36 | 0.3954 | 0.4138 | 0.956 | 0.3392 |
| UM:time36 | 1.832 | 0.8111 | 2.259 | 0.0239 |
| W:time36 | -1.792 | 0.7916 | -2.264 | 0.0236 |
| RP:time38 | 3.353 | 0.641 | 5.23 | <.0001 |
| TB:time38 | -0.0176 | 0.4625 | -0.038 | 0.9694 |
| UM:time38 | 2.228 | 0.7995 | 2.786 | 0.0053 |
| W:time38 | -1.253 | 0.6807 | -1.841 | 0.0656 |
| RP:time40 | 3.689 | 0.6521 | 5.658 | <.0001 |
| TB:time40 | 0.6295 | 0.4516 | 1.394 | 0.1633 |
| UM:time40 | 3.044 | 0.79 | 3.853 | 0.000117 |
| W:time40 | -0.9162 | 0.6911 | -1.326 | 0.1849 |
| RP:time42 | 3.402 | 0.6404 | 5.311 | <.0001 |
| TB:time42 | 0.2236 | 0.441 | 0.507 | 0.6121 |
| UM:time42 | 2.841 | 0.7771 | 3.656 | 0.0002 |
| W:time42 | -15.78 | 824.9 | -0.019 | 0.9847 |
| RP:time44 | 3.502 | 0.6566 | 5.335 | <.0001 |
| TB:time44 | 0.492 | 0.4658 | 1.056 | 0.2908 |
| UM:time44 | 3.246 | 0.7885 | 4.117 | <.0001 |
| W:time44 | -0.1692 | 0.5375 | -0.315 | 0.7529 |
| RP:time46 | 2.872 | 0.6319 | 4.545 | <.0001 |
| TB:time46 | -0.4683 | 0.4497 | -1.041 | 0.2976 |
| UM:time46 | 2.048 | 0.7835 | 2.613 | 0.0089 |
| W:time46 | -2.907 | 1.059 | -2.744 | 0.006 |
| RP:time48 | 3.347 | 0.6561 | 5.101 | <.0001 |
| TB:time48 | -0.409 | 0.5515 | -0.742 | 0.4583 |
| UM:time48 | 2.711 | 0.798 | 3.398 | 0.0006 |
| W:time48 | -1.082 | 0.6921 | -1.564 | 0.1178 |
| RP:time50 | 3.062 | 0.6869 | 4.458 | <.0001 |
| TB:time50 | 0.1267 | 0.5335 | 0.238 | 0.8122 |
| UM:time50 | 1.171 | 1.052 | 1.113 | 0.2656 |
| W:time50 | -1.333 | 0.8189 | -1.627 | 0.1036 |
| RP:time52 | 3.145 | 0.6705 | 4.691 | <.0001 |
| TB:time52 | -0.5376 | 0.589 | -0.913 | 0.3613 |
| UM:time52 | 1.892 | 0.8898 | 2.126 | 0.0334 |
| W:time52 | -15.92 | 948.1 | -0.017 | 0.9866 |
| RP:time54 | 3.138 | 0.6937 | 4.524 | <.0001 |
| TB:time54 | -1.832 | 1.079 | -1.699 | 0.0893 |
| UM:time54 | 3.011 | 0.8327 | 3.616 | 0.0002 |
| W:time54 | -1.914 | 1.086 | -1.762 | 0.0781 |
