# Supplementary material for: Escaping and repairing behaviors of the termite Odontotermes formosanus (Blattodea: Termitidae) in response to disturbance
Source: PeerJ. 2018 Mar 16;6:e4513. doi: 10.7717/peerj.4513 (PMC5858535; doi:10.7717/peerj.4513)
Supplement: Table S4 [file peerj-06-4513-s004.docx]

Table S4. Generalized linear mixed-effect models were built to compare the number of termite individuals that showed different behaviors after the mud tube had been damaged. Summary of post-hoc comparisons (Tukey’s Honestly Significant Difference tests) among the number of termites that exhibited downward moving (DM), repairing (RP), turning/backward moving (TB), upward moving (UM), and wandering (W) at each time interval are shown. The number in the “Time intervals” column indicates the time (minute) after tubes were damaged

a) Damaged mud tubes were completely repaired within 22 min:

| **Time intervals** | **Pairwise comparison** | **Estimate** | **SE** | **Z ratio** | ***P*** |
| --- | --- | --- | --- | --- | --- |
| 0 | DM-RP | 3.10338 | 0.545429 | 5.69 | <.0001 |
|  | DM-TB | 1.055423 | 0.288337 | 3.66 | 0.0023 |
|  | DM-UM | 3.837887 | 0.764209 | 5.022 | <.0001 |
|  | DM-W | 0.745174 | 0.295133 | 2.525 | 0.0851 |
|  | RP-TB | -2.04796 | 0.558412 | -3.667 | 0.0023 |
|  | RP-UM | 0.734507 | 0.891106 | 0.824 | 0.9232 |
|  | RP-W | -2.35821 | 0.561199 | -4.202 | 0.0003 |
|  | TB-UM | 2.782464 | 0.737506 | 3.773 | 0.0015 |
|  | TB-W | -0.31025 | 0.349395 | -0.888 | 0.9015 |
|  | UM-W | -3.09271 | 0.783874 | -3.945 | 0.0008 |
|  |  |  |  |  |  |
| 2 | DM-RP | 1.138751 | 0.234637 | 4.853 | <.0001 |
|  | DM-TB | 2.115849 | 0.288425 | 7.336 | <.0001 |
|  | DM-UM | 2.279477 | 0.346913 | 6.571 | <.0001 |
|  | DM-W | 2.796934 | 0.361418 | 7.739 | <.0001 |
|  | RP-TB | 0.977097 | 0.286009 | 3.416 | 0.0057 |
|  | RP-UM | 1.140726 | 0.316748 | 3.601 | 0.0029 |
|  | RP-W | 1.658182 | 0.358316 | 4.628 | <.0001 |
|  | TB-UM | 0.163629 | 0.304408 | 0.538 | 0.9834 |
|  | TB-W | 0.681085 | 0.421915 | 1.614 | 0.4881 |
|  | UM-W | 0.517456 | 0.454616 | 1.138 | 0.7862 |
|  |  |  |  |  |  |
| 4 | DM-RP | 1.225887 | 0.225161 | 5.444 | <.0001 |
|  | DM-TB | 2.066705 | 0.264129 | 7.825 | <.0001 |
|  | DM-UM | 2.700799 | 0.351105 | 7.692 | <.0001 |
|  | DM-W | 3.569189 | 0.411705 | 8.669 | <.0001 |
|  | RP-TB | 0.840818 | 0.257808 | 3.261 | 0.0098 |
|  | RP-UM | 1.474913 | 0.318346 | 4.633 | <.0001 |
|  | RP-W | 2.343302 | 0.406641 | 5.763 | <.0001 |
|  | TB-UM | 0.634095 | 0.29088 | 2.18 | 0.1873 |
|  | TB-W | 1.502484 | 0.453774 | 3.311 | 0.0083 |
|  | UM-W | 0.86839 | 0.500925 | 1.734 | 0.4132 |
|  |  |  |  |  |  |
| 6 | DM-RP | 1.286752 | 0.22208 | 5.794 | <.0001 |
|  | DM-TB | 2.241113 | 0.264662 | 8.468 | <.0001 |
|  | DM-UM | 2.674361 | 0.339436 | 7.879 | <.0001 |
|  | DM-W | 3.834914 | 0.427919 | 8.962 | <.0001 |
|  | RP-TB | 0.954361 | 0.257348 | 3.708 | 0.0019 |
|  | RP-UM | 1.387609 | 0.304577 | 4.556 | 0.0001 |
|  | RP-W | 2.548162 | 0.422435 | 6.032 | <.0001 |
|  | TB-UM | 0.433248 | 0.278737 | 1.554 | 0.527 |
|  | TB-W | 1.593801 | 0.469751 | 3.393 | 0.0062 |
|  | UM-W | 1.160553 | 0.507287 | 2.288 | 0.1487 |
|  |  |  |  |  |  |
| 8 | DM-RP | 1.187563 | 0.224015 | 5.301 | <.0001 |
|  | DM-TB | 2.414688 | 0.28376 | 8.51 | <.0001 |
|  | DM-UM | 1.92274 | 0.311984 | 6.163 | <.0001 |
|  | DM-W | 3.575326 | 0.411678 | 8.685 | <.0001 |
|  | RP-TB | 1.227125 | 0.277027 | 4.43 | 0.0001 |
|  | RP-UM | 0.735177 | 0.273729 | 2.686 | 0.0561 |
|  | RP-W | 2.387763 | 0.406027 | 5.881 | <.0001 |
|  | TB-UM | -0.49195 | 0.26356 | -1.867 | 0.3355 |
|  | TB-W | 1.160637 | 0.465491 | 2.493 | 0.0921 |
|  | UM-W | 1.652586 | 0.474342 | 3.484 | 0.0045 |
|  |  |  |  |  |  |
| 10 | DM-RP | 1.142186 | 0.225038 | 5.076 | <.0001 |
|  | DM-TB | 2.507616 | 0.295309 | 8.491 | <.0001 |
|  | DM-UM | 1.509687 | 0.301974 | 4.999 | <.0001 |
|  | DM-W | 4.095452 | 0.509361 | 8.04 | <.0001 |
|  | RP-TB | 1.36543 | 0.288891 | 4.726 | <.0001 |
|  | RP-UM | 0.367501 | 0.262313 | 1.401 | 0.627 |
|  | RP-W | 2.953266 | 0.504831 | 5.85 | <.0001 |
|  | TB-UM | -0.99793 | 0.263769 | -3.783 | 0.0015 |
|  | TB-W | 1.587836 | 0.559382 | 2.839 | 0.0366 |
|  | UM-W | 2.585765 | 0.555331 | 4.656 | <.0001 |
|  |  |  |  |  |  |
| 12 | DM-RP | 0.841139 | 0.223846 | 3.758 | 0.0016 |
|  | DM-TB | 2.461634 | 0.307304 | 8.01 | <.0001 |
|  | DM-UM | 0.987422 | 0.295741 | 3.339 | 0.0075 |
|  | DM-W | 3.909859 | 0.510046 | 7.666 | <.0001 |
|  | RP-TB | 1.620495 | 0.297987 | 5.438 | <.0001 |
|  | RP-UM | 0.146282 | 0.25138 | 0.582 | 0.9778 |
|  | RP-W | 3.068719 | 0.503649 | 6.093 | <.0001 |
|  | TB-UM | -1.47421 | 0.267808 | -5.505 | <.0001 |
|  | TB-W | 1.448224 | 0.565224 | 2.562 | 0.0775 |
|  | UM-W | 2.922437 | 0.551371 | 5.3 | <.0001 |
|  |  |  |  |  |  |
| 14 | DM-RP | 1.323332 | 0.246241 | 5.374 | <.0001 |
|  | DM-TB | 2.279946 | 0.319035 | 7.146 | <.0001 |
|  | DM-UM | 1.383642 | 0.321178 | 4.308 | 0.0002 |
|  | DM-W | 4.415941 | 0.631148 | 6.997 | <.0001 |
|  | RP-TB | 0.956614 | 0.323842 | 2.954 | 0.0261 |
|  | RP-UM | 0.06031 | 0.295051 | 0.204 | 0.9996 |
|  | RP-W | 3.092609 | 0.632987 | 4.886 | <.0001 |
|  | TB-UM | -0.8963 | 0.303037 | -2.958 | 0.0258 |
|  | TB-W | 2.135995 | 0.681821 | 3.133 | 0.0149 |
|  | UM-W | 3.032299 | 0.675478 | 4.489 | 0.0001 |
|  |  |  |  |  |  |
| 16 | DM-RP | 1.066956 | 0.246513 | 4.328 | 0.0001 |
|  | DM-TB | 1.972315 | 0.311952 | 6.322 | <.0001 |
|  | DM-UM | 0.834425 | 0.311618 | 2.678 | 0.0573 |
|  | DM-W | 3.925735 | 0.56156 | 6.991 | <.0001 |
|  | RP-TB | 0.905359 | 0.313558 | 2.887 | 0.0318 |
|  | RP-UM | -0.23253 | 0.280927 | -0.828 | 0.9221 |
|  | RP-W | 2.858778 | 0.561773 | 5.089 | <.0001 |
|  | TB-UM | -1.13789 | 0.281234 | -4.046 | 0.0005 |
|  | TB-W | 1.953419 | 0.612529 | 3.189 | 0.0124 |
|  | UM-W | 3.09131 | 0.604155 | 5.117 | <.0001 |
|  |  |  |  |  |  |
| 18 | DM-RP | 0.84233 | 0.269136 | 3.13 | 0.0151 |
|  | DM-TB | 2.163796 | 0.391709 | 5.524 | <.0001 |
|  | DM-UM | 0.458788 | 0.322799 | 1.421 | 0.6139 |
|  | DM-W | 4.861035 | 1.038807 | 4.679 | <.0001 |
|  | RP-TB | 1.321466 | 0.394715 | 3.348 | 0.0073 |
|  | RP-UM | -0.38354 | 0.294848 | -1.301 | 0.6907 |
|  | RP-W | 4.018706 | 1.039542 | 3.866 | 0.001 |
|  | TB-UM | -1.70501 | 0.361988 | -4.71 | <.0001 |
|  | TB-W | 2.69724 | 1.088978 | 2.477 | 0.0959 |
|  | UM-W | 4.402248 | 1.060905 | 4.15 | 0.0003 |
|  |  |  |  |  |  |
| 20 | DM-RP | 0.838034 | 0.419195 | 1.999 | 0.2663 |
|  | DM-TB | 2.445698 | 0.772591 | 3.166 | 0.0134 |
|  | DM-UM | 0.570298 | 0.451616 | 1.263 | 0.7141 |
|  | DM-W | 19.03717 | 1786.826 | 0.011 | 1 |
|  | RP-TB | 1.607664 | 0.793648 | 2.026 | 0.2536 |
|  | RP-UM | -0.26774 | 0.461408 | -0.58 | 0.978 |
|  | RP-W | 18.19913 | 1786.826 | 0.01 | 1 |
|  | TB-UM | -1.8754 | 0.770774 | -2.433 | 0.1065 |
|  | TB-W | 16.59147 | 1786.826 | 0.009 | 1 |
|  | UM-W | 18.46687 | 1786.826 | 0.01 | 1 |
|  |  |  |  |  |  |
| 22 | DM-RP | 1.004116 | 0.379044 | 2.649 | 0.0618 |
|  | DM-TB | 3.527805 | 1.040962 | 3.389 | 0.0063 |
|  | DM-UM | 0.602894 | 0.408843 | 1.475 | 0.579 |
|  | DM-W | 19.40786 | 1770.272 | 0.011 | 1 |
|  | RP-TB | 2.523689 | 1.05605 | 2.39 | 0.118 |
|  | RP-UM | -0.40122 | 0.418034 | -0.96 | 0.8731 |
|  | RP-W | 18.40375 | 1770.272 | 0.01 | 1 |
|  | TB-UM | -2.92491 | 1.036678 | -2.821 | 0.0385 |
|  | TB-W | 15.88006 | 1770.272 | 0.009 | 1 |
|  | UM-W | 18.80497 | 1770.272 | 0.011 | 1 |

b) Damaged mud tubes were completely repaired between 24 and 40 min:

| **Time intervals** | **Pairwise comparison** | **Estimate** | **SE** | **Z value** | ***P*** |
| --- | --- | --- | --- | --- | --- |
| 0 | DM-RP | 2.115721 | 0.255058 | 8.295 | <.0001 |
|  | DM-TB | 1.357965 | 0.203221 | 6.682 | <.0001 |
|  | DM-UM | 2.358769 | 0.307838 | 7.662 | <.0001 |
|  | DM-W | 0.76319 | 0.202348 | 3.772 | 0.0015 |
|  | RP-TB | -0.75776 | 0.283836 | -2.67 | 0.0585 |
|  | RP-UM | 0.243048 | 0.364099 | 0.668 | 0.9633 |
|  | RP-W | -1.35253 | 0.287142 | -4.71 | <.0001 |
|  | TB-UM | 1.000804 | 0.295433 | 3.388 | 0.0063 |
|  | TB-W | -0.59478 | 0.217462 | -2.735 | 0.049 |
|  | UM-W | -1.59558 | 0.30906 | -5.163 | <.0001 |
|  |  |  |  |  |  |
| 2 | DM-RP | 0.856965 | 0.159828 | 5.362 | <.0001 |
|  | DM-TB | 1.465486 | 0.195041 | 7.514 | <.0001 |
|  | DM-UM | 1.400759 | 0.235783 | 5.941 | <.0001 |
|  | DM-W | 1.966953 | 0.24215 | 8.123 | <.0001 |
|  | RP-TB | 0.608521 | 0.199886 | 3.044 | 0.0198 |
|  | RP-UM | 0.543794 | 0.236772 | 2.297 | 0.1458 |
|  | RP-W | 1.109988 | 0.250589 | 4.43 | 0.0001 |
|  | TB-UM | -0.06473 | 0.216785 | -0.299 | 0.9983 |
|  | TB-W | 0.501467 | 0.252725 | 1.984 | 0.2736 |
|  | UM-W | 0.566194 | 0.276009 | 2.051 | 0.2416 |
|  |  |  |  |  |  |
| 4 | DM-RP | 0.884786 | 0.146809 | 6.027 | <.0001 |
|  | DM-TB | 1.380137 | 0.174318 | 7.917 | <.0001 |
|  | DM-UM | 2.079168 | 0.249242 | 8.342 | <.0001 |
|  | DM-W | 2.774365 | 0.277159 | 10.01 | <.0001 |
|  | RP-TB | 0.495351 | 0.175232 | 2.827 | 0.0379 |
|  | RP-UM | 1.194382 | 0.246973 | 4.836 | <.0001 |
|  | RP-W | 1.88958 | 0.281747 | 6.707 | <.0001 |
|  | TB-UM | 0.699031 | 0.2197 | 3.182 | 0.0127 |
|  | TB-W | 1.394229 | 0.277117 | 5.031 | <.0001 |
|  | UM-W | 0.695197 | 0.321384 | 2.163 | 0.1938 |
|  |  |  |  |  |  |
| 6 | DM-RP | 0.823778 | 0.140184 | 5.876 | <.0001 |
|  | DM-TB | 1.94162 | 0.193048 | 10.058 | <.0001 |
|  | DM-UM | 1.836072 | 0.232359 | 7.902 | <.0001 |
|  | DM-W | 2.76312 | 0.264563 | 10.444 | <.0001 |
|  | RP-TB | 1.117842 | 0.191028 | 5.852 | <.0001 |
|  | RP-UM | 1.012295 | 0.227528 | 4.449 | 0.0001 |
|  | RP-W | 1.939342 | 0.267326 | 7.255 | <.0001 |
|  | TB-UM | -0.10555 | 0.218686 | -0.483 | 0.989 |
|  | TB-W | 0.8215 | 0.278671 | 2.948 | 0.0265 |
|  | UM-W | 0.927048 | 0.298562 | 3.105 | 0.0163 |
|  |  |  |  |  |  |
| 8 | DM-RP | 0.923561 | 0.132948 | 6.947 | <.0001 |
|  | DM-TB | 1.636033 | 0.165039 | 9.913 | <.0001 |
|  | DM-UM | 2.196689 | 0.233505 | 9.407 | <.0001 |
|  | DM-W | 3.354742 | 0.290451 | 11.55 | <.0001 |
|  | RP-TB | 0.712472 | 0.161124 | 4.422 | 0.0001 |
|  | RP-UM | 1.273128 | 0.227601 | 5.594 | <.0001 |
|  | RP-W | 2.431181 | 0.292114 | 8.323 | <.0001 |
|  | TB-UM | 0.560656 | 0.199508 | 2.81 | 0.0397 |
|  | TB-W | 1.718709 | 0.288917 | 5.949 | <.0001 |
|  | UM-W | 1.158052 | 0.324836 | 3.565 | 0.0033 |
|  |  |  |  |  |  |
| 10 | DM-RP | 0.755063 | 0.13709 | 5.508 | <.0001 |
|  | DM-TB | 1.678859 | 0.178135 | 9.425 | <.0001 |
|  | DM-UM | 1.32179 | 0.21579 | 6.125 | <.0001 |
|  | DM-W | 2.720253 | 0.257805 | 10.552 | <.0001 |
|  | RP-TB | 0.923795 | 0.174155 | 5.304 | <.0001 |
|  | RP-UM | 0.566727 | 0.209087 | 2.71 | 0.0524 |
|  | RP-W | 1.96519 | 0.259433 | 7.575 | <.0001 |
|  | TB-UM | -0.35707 | 0.187334 | -1.906 | 0.314 |
|  | TB-W | 1.041394 | 0.262339 | 3.97 | 0.0007 |
|  | UM-W | 1.398463 | 0.280024 | 4.994 | <.0001 |
|  |  |  |  |  |  |
| 12 | DM-RP | 0.564848 | 0.136073 | 4.151 | 0.0003 |
|  | DM-TB | 1.596493 | 0.179614 | 8.888 | <.0001 |
|  | DM-UM | 1.09407 | 0.213669 | 5.12 | <.0001 |
|  | DM-W | 2.75024 | 0.268557 | 10.241 | <.0001 |
|  | RP-TB | 1.031645 | 0.17321 | 5.956 | <.0001 |
|  | RP-UM | 0.529222 | 0.204815 | 2.584 | 0.0733 |
|  | RP-W | 2.185392 | 0.268529 | 8.138 | <.0001 |
|  | TB-UM | -0.50242 | 0.184751 | -2.719 | 0.0512 |
|  | TB-W | 1.153747 | 0.272821 | 4.229 | 0.0002 |
|  | UM-W | 1.65617 | 0.287371 | 5.763 | <.0001 |
|  |  |  |  |  |  |
| 14 | DM-RP | 0.619724 | 0.132633 | 4.672 | <.0001 |
|  | DM-TB | 1.518085 | 0.169505 | 8.956 | <.0001 |
|  | DM-UM | 1.462007 | 0.217715 | 6.715 | <.0001 |
|  | DM-W | 3.906435 | 0.387237 | 10.088 | <.0001 |
|  | RP-TB | 0.898361 | 0.162441 | 5.53 | <.0001 |
|  | RP-UM | 0.842284 | 0.208829 | 4.033 | 0.0005 |
|  | RP-W | 3.286711 | 0.387108 | 8.49 | <.0001 |
|  | TB-UM | -0.05608 | 0.182177 | -0.308 | 0.9981 |
|  | TB-W | 2.38835 | 0.386745 | 6.176 | <.0001 |
|  | UM-W | 2.444427 | 0.403733 | 6.055 | <.0001 |
|  |  |  |  |  |  |
| 16 | DM-RP | 0.575598 | 0.131364 | 4.382 | 0.0001 |
|  | DM-TB | 1.679194 | 0.175149 | 9.587 | <.0001 |
|  | DM-UM | 1.324226 | 0.214117 | 6.185 | <.0001 |
|  | DM-W | 3.919592 | 0.387171 | 10.124 | <.0001 |
|  | RP-TB | 1.103596 | 0.167536 | 6.587 | <.0001 |
|  | RP-UM | 0.748628 | 0.20443 | 3.662 | 0.0023 |
|  | RP-W | 3.343993 | 0.3867 | 8.648 | <.0001 |
|  | TB-UM | -0.35497 | 0.183441 | -1.935 | 0.2986 |
|  | TB-W | 2.240397 | 0.389278 | 5.755 | <.0001 |
|  | UM-W | 2.595366 | 0.401825 | 6.459 | <.0001 |
|  |  |  |  |  |  |
| 18 | DM-RP | 0.892284 | 0.1305 | 6.837 | <.0001 |
|  | DM-TB | 2.067425 | 0.179601 | 11.511 | <.0001 |
|  | DM-UM | 1.66058 | 0.214751 | 7.733 | <.0001 |
|  | DM-W | 3.84978 | 0.34031 | 11.313 | <.0001 |
|  | RP-TB | 1.175141 | 0.174899 | 6.719 | <.0001 |
|  | RP-UM | 0.768296 | 0.207377 | 3.705 | 0.002 |
|  | RP-W | 2.957495 | 0.341157 | 8.669 | <.0001 |
|  | TB-UM | -0.40685 | 0.191494 | -2.125 | 0.2095 |
|  | TB-W | 1.782354 | 0.346689 | 5.141 | <.0001 |
|  | UM-W | 2.189199 | 0.358905 | 6.1 | <.0001 |
|  |  |  |  |  |  |
| 20 | DM-RP | 0.883548 | 0.12951 | 6.822 | <.0001 |
|  | DM-TB | 2.031429 | 0.176523 | 11.508 | <.0001 |
|  | DM-UM | 1.520557 | 0.210907 | 7.21 | <.0001 |
|  | DM-W | 4.48455 | 0.436192 | 10.281 | <.0001 |
|  | RP-TB | 1.147881 | 0.171329 | 6.7 | <.0001 |
|  | RP-UM | 0.637009 | 0.20305 | 3.137 | 0.0147 |
|  | RP-W | 3.601002 | 0.436694 | 8.246 | <.0001 |
|  | TB-UM | -0.51087 | 0.184541 | -2.768 | 0.0447 |
|  | TB-W | 2.453121 | 0.440074 | 5.574 | <.0001 |
|  | UM-W | 2.963994 | 0.449158 | 6.599 | <.0001 |
|  |  |  |  |  |  |
| 22 | DM-RP | 0.719093 | 0.130005 | 5.531 | <.0001 |
|  | DM-TB | 1.766458 | 0.171853 | 10.279 | <.0001 |
|  | DM-UM | 1.242709 | 0.208844 | 5.95 | <.0001 |
|  | DM-W | 4.350986 | 0.436546 | 9.967 | <.0001 |
|  | RP-TB | 1.047365 | 0.165165 | 6.341 | <.0001 |
|  | RP-UM | 0.523616 | 0.199791 | 2.621 | 0.0666 |
|  | RP-W | 3.631893 | 0.436536 | 8.32 | <.0001 |
|  | TB-UM | -0.52375 | 0.17603 | -2.975 | 0.0244 |
|  | TB-W | 2.584528 | 0.437922 | 5.902 | <.0001 |
|  | UM-W | 3.108277 | 0.447902 | 6.94 | <.0001 |
|  |  |  |  |  |  |
| 24 | DM-RP | 0.751288 | 0.129301 | 5.81 | <.0001 |
|  | DM-TB | 1.870065 | 0.173829 | 10.758 | <.0001 |
|  | DM-UM | 1.125329 | 0.205867 | 5.466 | <.0001 |
|  | DM-W | 4.244175 | 0.408677 | 10.385 | <.0001 |
|  | RP-TB | 1.118777 | 0.167311 | 6.687 | <.0001 |
|  | RP-UM | 0.374041 | 0.196753 | 1.901 | 0.3166 |
|  | RP-W | 3.492887 | 0.408707 | 8.546 | <.0001 |
|  | TB-UM | -0.74474 | 0.175065 | -4.254 | 0.0002 |
|  | TB-W | 2.37411 | 0.411239 | 5.773 | <.0001 |
|  | UM-W | 3.118846 | 0.419571 | 7.433 | <.0001 |
|  |  |  |  |  |  |
| 26 | DM-RP | 0.657006 | 0.135017 | 4.866 | <.0001 |
|  | DM-TB | 1.745826 | 0.182879 | 9.546 | <.0001 |
|  | DM-UM | 0.856008 | 0.207962 | 4.116 | 0.0004 |
|  | DM-W | 4.568349 | 0.523284 | 8.73 | <.0001 |
|  | RP-TB | 1.08882 | 0.176807 | 6.158 | <.0001 |
|  | RP-UM | 0.199002 | 0.199082 | 1 | 0.8557 |
|  | RP-W | 3.911343 | 0.523345 | 7.474 | <.0001 |
|  | TB-UM | -0.88982 | 0.182369 | -4.879 | <.0001 |
|  | TB-W | 2.822523 | 0.526958 | 5.356 | <.0001 |
|  | UM-W | 3.71234 | 0.531264 | 6.988 | <.0001 |
|  |  |  |  |  |  |
| 28 | DM-RP | 0.768835 | 0.13924 | 5.522 | <.0001 |
|  | DM-TB | 1.780152 | 0.187936 | 9.472 | <.0001 |
|  | DM-UM | 1.131422 | 0.216499 | 5.226 | <.0001 |
|  | DM-W | 5.314601 | 0.722438 | 7.356 | <.0001 |
|  | RP-TB | 1.011318 | 0.185147 | 5.462 | <.0001 |
|  | RP-UM | 0.362587 | 0.210827 | 1.72 | 0.4216 |
|  | RP-W | 4.545766 | 0.723298 | 6.285 | <.0001 |
|  | TB-UM | -0.64873 | 0.196756 | -3.297 | 0.0087 |
|  | TB-W | 3.534449 | 0.726406 | 4.866 | <.0001 |
|  | UM-W | 4.183179 | 0.730735 | 5.725 | <.0001 |
|  |  |  |  |  |  |
| 30 | DM-RP | 0.684653 | 0.144618 | 4.734 | <.0001 |
|  | DM-TB | 1.890333 | 0.207684 | 9.102 | <.0001 |
|  | DM-UM | 0.798119 | 0.215038 | 3.712 | 0.0019 |
|  | DM-W | 5.077358 | 0.722911 | 7.023 | <.0001 |
|  | RP-TB | 1.20568 | 0.205556 | 5.865 | <.0001 |
|  | RP-UM | 0.113466 | 0.209794 | 0.541 | 0.9831 |
|  | RP-W | 4.392705 | 0.72394 | 6.068 | <.0001 |
|  | TB-UM | -1.09221 | 0.210975 | -5.177 | <.0001 |
|  | TB-W | 3.187025 | 0.731249 | 4.358 | 0.0001 |
|  | UM-W | 4.279239 | 0.729888 | 5.863 | <.0001 |
|  |  |  |  |  |  |
| 32 | DM-RP | 0.398816 | 0.158338 | 2.519 | 0.0865 |
|  | DM-TB | 2.465641 | 0.301899 | 8.167 | <.0001 |
|  | DM-UM | 0.641818 | 0.225403 | 2.847 | 0.0357 |
|  | DM-W | 3.946214 | 0.529331 | 7.455 | <.0001 |
|  | RP-TB | 2.066825 | 0.300613 | 6.875 | <.0001 |
|  | RP-UM | 0.243002 | 0.220639 | 1.101 | 0.8059 |
|  | RP-W | 3.547398 | 0.530877 | 6.682 | <.0001 |
|  | TB-UM | -1.82382 | 0.305005 | -5.98 | <.0001 |
|  | TB-W | 1.480573 | 0.579853 | 2.553 | 0.0793 |
|  | UM-W | 3.304395 | 0.539303 | 6.127 | <.0001 |
|  |  |  |  |  |  |
| 34 | DM-RP | 0.793261 | 0.257501 | 3.081 | 0.0176 |
|  | DM-TB | 2.396226 | 0.448111 | 5.347 | <.0001 |
|  | DM-UM | 1.635903 | 0.380292 | 4.302 | 0.0002 |
|  | DM-W | 18.24196 | 57.34862 | 0.318 | 0.9978 |
|  | RP-TB | 1.602965 | 0.469206 | 3.416 | 0.0057 |
|  | RP-UM | 0.842642 | 0.403293 | 2.089 | 0.2246 |
|  | RP-W | 17.4487 | 57.34905 | 0.304 | 0.9981 |
|  | TB-UM | -0.76032 | 0.524591 | -1.449 | 0.5956 |
|  | TB-W | 15.84574 | 57.34945 | 0.276 | 0.9987 |
|  | UM-W | 16.60606 | 57.34987 | 0.29 | 0.9985 |
|  |  |  |  |  |  |
| 36 | DM-RP | 0.970458 | 0.452429 | 2.145 | 0.2011 |
|  | DM-TB | 3.316008 | 1.04129 | 3.185 | 0.0126 |
|  | DM-UM | -0.00765 | 0.373623 | -0.02 | 1 |
|  | DM-W | 17.31141 | 68.84605 | 0.251 | 0.9991 |
|  | RP-TB | 2.345549 | 1.068881 | 2.194 | 0.1817 |
|  | RP-UM | -0.9781 | 0.444058 | -2.203 | 0.1786 |
|  | RP-W | 16.34095 | 68.84837 | 0.237 | 0.9993 |
|  | TB-UM | -3.32365 | 1.026596 | -3.238 | 0.0106 |
|  | TB-W | 13.99541 | 68.85637 | 0.203 | 0.9996 |
|  | UM-W | 17.31906 | 68.84733 | 0.252 | 0.9991 |
|  |  |  |  |  |  |
| 38 | DM-RP | 1.663751 | 0.442512 | 3.76 | 0.0016 |
|  | DM-TB | 2.777319 | 0.627953 | 4.423 | 0.0001 |
|  | DM-UM | 0.817553 | 0.350676 | 2.331 | 0.135 |
|  | DM-W | 18.00367 | 128.1641 | 0.14 | 0.9999 |
|  | RP-TB | 1.113569 | 0.705088 | 1.579 | 0.5107 |
|  | RP-UM | -0.8462 | 0.474522 | -1.783 | 0.3833 |
|  | RP-W | 16.33992 | 128.1649 | 0.127 | 0.9999 |
|  | TB-UM | -1.95977 | 0.633073 | -3.096 | 0.0168 |
|  | TB-W | 15.22635 | 128.1689 | 0.119 | 1 |
|  | UM-W | 17.18612 | 128.1645 | 0.134 | 0.9999 |
|  |  |  |  |  |  |
| 40 | DM-RP | 1.635755 | 0.396202 | 4.129 | 0.0004 |
|  | DM-TB | 3.406634 | 0.743904 | 4.579 | <.0001 |
|  | DM-UM | 1.468446 | 0.372786 | 3.939 | 0.0008 |
|  | DM-W | 18.14588 | 97.09878 | 0.187 | 0.9997 |
|  | RP-TB | 1.770879 | 0.794769 | 2.228 | 0.1692 |
|  | RP-UM | -0.16731 | 0.465415 | -0.359 | 0.9964 |
|  | RP-W | 16.51013 | 97.09969 | 0.17 | 0.9998 |
|  | TB-UM | -1.93819 | 0.768197 | -2.523 | 0.0855 |
|  | TB-W | 14.73925 | 97.09753 | 0.152 | 0.9999 |
|  | UM-W | 16.67743 | 97.09909 | 0.172 | 0.9998 |

c) Damaged mud tubes were completely repaired more than 42 min:

| **Time intervals** | **Pairwise comparison** | **Estimate** | **SE** | **Z value** | ***P*** |
| --- | --- | --- | --- | --- | --- |
| 0 | DM-RP | 2.349669 | 0.627486 | 3.745 | 0.0017 |
|  | DM-TB | 0.745105 | 0.319674 | 2.331 | 0.1351 |
|  | DM-UM | 3.391355 | 0.753499 | 4.501 | 0.0001 |
|  | DM-W | 0.753279 | 0.42232 | 1.784 | 0.3831 |
|  | RP-TB | -1.60456 | 0.640976 | -2.503 | 0.0898 |
|  | RP-UM | 1.041685 | 0.974999 | 1.068 | 0.8228 |
|  | RP-W | -1.59639 | 0.655885 | -2.434 | 0.1063 |
|  | TB-UM | 2.646249 | 0.789394 | 3.352 | 0.0072 |
|  | TB-W | 0.008174 | 0.428486 | 0.019 | 1 |
|  | UM-W | -2.63808 | 0.884827 | -2.981 | 0.024 |
|  |  |  |  |  |  |
| 2 | DM-RP | 0.969936 | 0.357541 | 2.713 | 0.0521 |
|  | DM-TB | 0.420448 | 0.2728 | 1.541 | 0.5355 |
|  | DM-UM | 2.454748 | 0.479425 | 5.12 | <.0001 |
|  | DM-W | 0.487305 | 0.388948 | 1.253 | 0.7201 |
|  | RP-TB | -0.54949 | 0.353026 | -1.557 | 0.5255 |
|  | RP-UM | 1.484812 | 0.595197 | 2.495 | 0.0918 |
|  | RP-W | -0.48263 | 0.380342 | -1.269 | 0.7104 |
|  | TB-UM | 2.0343 | 0.514695 | 3.952 | 0.0007 |
|  | TB-W | 0.066857 | 0.369063 | 0.181 | 0.9998 |
|  | UM-W | -1.96744 | 0.652215 | -3.017 | 0.0215 |
|  |  |  |  |  |  |
| 4 | DM-RP | 1.017161 | 0.287598 | 3.537 | 0.0037 |
|  | DM-TB | 1.897343 | 0.329377 | 5.76 | <.0001 |
|  | DM-UM | 2.177603 | 0.338516 | 6.433 | <.0001 |
|  | DM-W | 1.382011 | 0.386979 | 3.571 | 0.0033 |
|  | RP-TB | 0.880182 | 0.367499 | 2.395 | 0.1166 |
|  | RP-UM | 1.160441 | 0.464072 | 2.501 | 0.0905 |
|  | RP-W | 0.364849 | 0.345675 | 1.055 | 0.8292 |
|  | TB-UM | 0.28026 | 0.452997 | 0.619 | 0.9722 |
|  | TB-W | -0.51533 | 0.436154 | -1.182 | 0.762 |
|  | UM-W | -0.79559 | 0.574528 | -1.385 | 0.6375 |
|  |  |  |  |  |  |
| 6 | DM-RP | 0.952159 | 0.265271 | 3.589 | 0.0031 |
|  | DM-TB | 1.454233 | 0.254059 | 5.724 | <.0001 |
|  | DM-UM | 3.120994 | 0.418307 | 7.461 | <.0001 |
|  | DM-W | 2.178857 | 0.431244 | 5.052 | <.0001 |
|  | RP-TB | 0.502074 | 0.288832 | 1.738 | 0.4103 |
|  | RP-UM | 2.168835 | 0.517747 | 4.189 | 0.0003 |
|  | RP-W | 1.226698 | 0.384736 | 3.188 | 0.0125 |
|  | TB-UM | 1.666761 | 0.475717 | 3.504 | 0.0042 |
|  | TB-W | 0.724624 | 0.432622 | 1.675 | 0.4495 |
|  | UM-W | -0.94214 | 0.656768 | -1.435 | 0.6053 |
|  |  |  |  |  |  |
| 8 | DM-RP | 0.793634 | 0.258195 | 3.074 | 0.018 |
|  | DM-TB | 1.222903 | 0.237267 | 5.154 | <.0001 |
|  | DM-UM | 2.794093 | 0.375267 | 7.446 | <.0001 |
|  | DM-W | 2.003445 | 0.414668 | 4.831 | <.0001 |
|  | RP-TB | 0.429269 | 0.267084 | 1.607 | 0.4927 |
|  | RP-UM | 2.000459 | 0.479652 | 4.171 | 0.0003 |
|  | RP-W | 1.209811 | 0.360777 | 3.353 | 0.0071 |
|  | TB-UM | 1.57119 | 0.428678 | 3.665 | 0.0023 |
|  | TB-W | 0.780542 | 0.405899 | 1.923 | 0.305 |
|  | UM-W | -0.79065 | 0.618895 | -1.278 | 0.7051 |
|  |  |  |  |  |  |
| 10 | DM-RP | 0.770586 | 0.252096 | 3.057 | 0.019 |
|  | DM-TB | 1.343878 | 0.238239 | 5.641 | <.0001 |
|  | DM-UM | 3.199775 | 0.417571 | 7.663 | <.0001 |
|  | DM-W | 2.016103 | 0.407079 | 4.953 | <.0001 |
|  | RP-TB | 0.573292 | 0.264689 | 2.166 | 0.1927 |
|  | RP-UM | 2.429189 | 0.511733 | 4.747 | <.0001 |
|  | RP-W | 1.245517 | 0.349554 | 3.563 | 0.0034 |
|  | TB-UM | 1.855897 | 0.468128 | 3.965 | 0.0007 |
|  | TB-W | 0.672225 | 0.400445 | 1.679 | 0.4472 |
|  | UM-W | -1.18367 | 0.641648 | -1.845 | 0.3477 |
|  |  |  |  |  |  |
| 12 | DM-RP | 0.288647 | 0.255189 | 1.131 | 0.7901 |
|  | DM-TB | 1.150083 | 0.259734 | 4.428 | 0.0001 |
|  | DM-UM | 2.123661 | 0.339389 | 6.257 | <.0001 |
|  | DM-W | 2.192907 | 0.472427 | 4.642 | <.0001 |
|  | RP-TB | 0.861437 | 0.27435 | 3.14 | 0.0146 |
|  | RP-UM | 1.835014 | 0.444066 | 4.132 | 0.0003 |
|  | RP-W | 1.904261 | 0.417332 | 4.563 | <.0001 |
|  | TB-UM | 0.973578 | 0.404478 | 2.407 | 0.1134 |
|  | TB-W | 1.042824 | 0.470605 | 2.216 | 0.1737 |
|  | UM-W | 0.069246 | 0.634776 | 0.109 | 1 |
|  |  |  |  |  |  |
| 14 | DM-RP | 0.562811 | 0.256792 | 2.192 | 0.1827 |
|  | DM-TB | 1.26342 | 0.2534 | 4.986 | <.0001 |
|  | DM-UM | 2.565348 | 0.36673 | 6.995 | <.0001 |
|  | DM-W | 2.346999 | 0.470737 | 4.986 | <.0001 |
|  | RP-TB | 0.700608 | 0.2757 | 2.541 | 0.0817 |
|  | RP-UM | 2.002537 | 0.469567 | 4.265 | 0.0002 |
|  | RP-W | 1.784188 | 0.420196 | 4.246 | 0.0002 |
|  | TB-UM | 1.301928 | 0.427586 | 3.045 | 0.0197 |
|  | TB-W | 1.08358 | 0.468825 | 2.311 | 0.1412 |
|  | UM-W | -0.21835 | 0.651018 | -0.335 | 0.9973 |
|  |  |  |  |  |  |
| 16 | DM-RP | -0.18381 | 0.270669 | -0.679 | 0.961 |
|  | DM-TB | 0.726211 | 0.280435 | 2.59 | 0.0722 |
|  | DM-UM | 1.746092 | 0.362666 | 4.815 | <.0001 |
|  | DM-W | 1.815372 | 0.498531 | 3.641 | 0.0025 |
|  | RP-TB | 0.910017 | 0.282466 | 3.222 | 0.0112 |
|  | RP-UM | 1.929898 | 0.454834 | 4.243 | 0.0002 |
|  | RP-W | 1.999178 | 0.439146 | 4.552 | 0.0001 |
|  | TB-UM | 1.019881 | 0.419908 | 2.429 | 0.1076 |
|  | TB-W | 1.08916 | 0.493147 | 2.209 | 0.1764 |
|  | UM-W | 0.069279 | 0.65561 | 0.106 | 1 |
|  |  |  |  |  |  |
| 18 | DM-RP | -0.05852 | 0.267957 | -0.218 | 0.9995 |
|  | DM-TB | 0.744647 | 0.270198 | 2.756 | 0.0463 |
|  | DM-UM | 2.293477 | 0.41169 | 5.571 | <.0001 |
|  | DM-W | 2.075376 | 0.520293 | 3.989 | 0.0006 |
|  | RP-TB | 0.803171 | 0.276397 | 2.906 | 0.0301 |
|  | RP-UM | 2.352 | 0.497072 | 4.732 | <.0001 |
|  | RP-W | 2.133899 | 0.466117 | 4.578 | <.0001 |
|  | TB-UM | 1.54883 | 0.460819 | 3.361 | 0.007 |
|  | TB-W | 1.330728 | 0.513258 | 2.593 | 0.0717 |
|  | UM-W | -0.2181 | 0.702612 | -0.31 | 0.998 |
|  |  |  |  |  |  |
| 20 | DM-RP | 0.000306 | 0.260015 | 0.001 | 1 |
|  | DM-TB | 1.081099 | 0.280992 | 3.847 | 0.0011 |
|  | DM-UM | 1.754505 | 0.333746 | 5.257 | <.0001 |
|  | DM-W | 2.075369 | 0.494342 | 4.198 | 0.0003 |
|  | RP-TB | 1.080793 | 0.287933 | 3.754 | 0.0016 |
|  | RP-UM | 1.754199 | 0.43536 | 4.029 | 0.0005 |
|  | RP-W | 2.075063 | 0.437601 | 4.742 | <.0001 |
|  | TB-UM | 0.673407 | 0.406146 | 1.658 | 0.4602 |
|  | TB-W | 0.99427 | 0.497802 | 1.997 | 0.2672 |
|  | UM-W | 0.320864 | 0.643416 | 0.499 | 0.9875 |
|  |  |  |  |  |  |
| 22 | DM-RP | 0.021528 | 0.265559 | 0.081 | 1 |
|  | DM-TB | 0.911768 | 0.274797 | 3.318 | 0.0081 |
|  | DM-UM | 2.778934 | 0.473223 | 5.872 | <.0001 |
|  | DM-W | 2.155253 | 0.519039 | 4.152 | 0.0003 |
|  | RP-TB | 0.89024 | 0.283165 | 3.144 | 0.0144 |
|  | RP-UM | 2.757405 | 0.550284 | 5.011 | <.0001 |
|  | RP-W | 2.133724 | 0.466094 | 4.578 | <.0001 |
|  | TB-UM | 1.867166 | 0.521413 | 3.581 | 0.0032 |
|  | TB-W | 1.243485 | 0.516912 | 2.406 | 0.1137 |
|  | UM-W | -0.62368 | 0.741197 | -0.841 | 0.9177 |
|  |  |  |  |  |  |
| 24 | DM-RP | -0.68274 | 0.284182 | -2.402 | 0.1146 |
|  | DM-TB | 0.772952 | 0.337401 | 2.291 | 0.1477 |
|  | DM-UM | 2.188123 | 0.486144 | 4.501 | 0.0001 |
|  | DM-W | 1.276763 | 0.489744 | 2.607 | 0.0691 |
|  | RP-TB | 1.455696 | 0.322462 | 4.514 | 0.0001 |
|  | RP-UM | 2.870866 | 0.548345 | 5.236 | <.0001 |
|  | RP-W | 1.959506 | 0.41613 | 4.709 | <.0001 |
|  | TB-UM | 1.41517 | 0.545737 | 2.593 | 0.0716 |
|  | TB-W | 0.50381 | 0.501205 | 1.005 | 0.8531 |
|  | UM-W | -0.91136 | 0.712356 | -1.279 | 0.704 |
|  |  |  |  |  |  |
| 26 | DM-RP | -0.65594 | 0.287338 | -2.283 | 0.1504 |
|  | DM-TB | 1.081079 | 0.378354 | 2.857 | 0.0347 |
|  | DM-UM | 1.466661 | 0.391807 | 3.743 | 0.0017 |
|  | DM-W | 1.7185 | 0.562262 | 3.056 | 0.019 |
|  | RP-TB | 1.73702 | 0.365391 | 4.754 | <.0001 |
|  | RP-UM | 2.122602 | 0.466985 | 4.545 | 0.0001 |
|  | RP-W | 2.374442 | 0.499669 | 4.752 | <.0001 |
|  | TB-UM | 0.385582 | 0.492677 | 0.783 | 0.9357 |
|  | TB-W | 0.637422 | 0.596004 | 1.069 | 0.8223 |
|  | UM-W | 0.251839 | 0.706633 | 0.356 | 0.9966 |
|  |  |  |  |  |  |
| 28 | DM-RP | -1.30518 | 0.319887 | -4.08 | 0.0004 |
|  | DM-TB | 0.426667 | 0.394301 | 1.082 | 0.8159 |
|  | DM-UM | 1.600644 | 0.508436 | 3.148 | 0.0142 |
|  | DM-W | 0.977029 | 0.551344 | 1.772 | 0.39 |
|  | RP-TB | 1.731851 | 0.350454 | 4.942 | <.0001 |
|  | RP-UM | 2.905828 | 0.547773 | 5.305 | <.0001 |
|  | RP-W | 2.282213 | 0.463138 | 4.928 | <.0001 |
|  | TB-UM | 1.173977 | 0.563265 | 2.084 | 0.2268 |
|  | TB-W | 0.550362 | 0.559111 | 0.984 | 0.8625 |
|  | UM-W | -0.62362 | 0.741207 | -0.841 | 0.9177 |
|  |  |  |  |  |  |
| 30 | DM-RP | -1.12343 | 0.306645 | -3.664 | 0.0023 |
|  | DM-TB | -0.12921 | 0.315928 | -0.409 | 0.9941 |
|  | DM-UM | 1.176251 | 0.417387 | 2.818 | 0.0388 |
|  | DM-W | 0.753146 | 0.489629 | 1.538 | 0.5375 |
|  | RP-TB | 0.994215 | 0.274687 | 3.619 | 0.0027 |
|  | RP-UM | 2.29968 | 0.473347 | 4.858 | <.0001 |
|  | RP-W | 1.876574 | 0.398185 | 4.713 | <.0001 |
|  | TB-UM | 1.305465 | 0.440345 | 2.965 | 0.0252 |
|  | TB-W | 0.88236 | 0.457486 | 1.929 | 0.302 |
|  | UM-W | -0.42311 | 0.646098 | -0.655 | 0.9658 |
|  |  |  |  |  |  |
| 32 | DM-RP | -0.30659 | 0.260685 | -1.176 | 0.7651 |
|  | DM-TB | 0.910092 | 0.285683 | 3.186 | 0.0126 |
|  | DM-UM | 1.834637 | 0.360496 | 5.089 | <.0001 |
|  | DM-W | 2.463737 | 0.595691 | 4.136 | 0.0003 |
|  | RP-TB | 1.216687 | 0.283884 | 4.286 | 0.0002 |
|  | RP-UM | 2.141232 | 0.450706 | 4.751 | <.0001 |
|  | RP-W | 2.770332 | 0.544973 | 5.083 | <.0001 |
|  | TB-UM | 0.924546 | 0.425278 | 2.174 | 0.1896 |
|  | TB-W | 1.553646 | 0.596336 | 2.605 | 0.0694 |
|  | UM-W | 0.6291 | 0.733285 | 0.858 | 0.9121 |
|  |  |  |  |  |  |
| 34 | DM-RP | -0.0819 | 0.259005 | -0.316 | 0.9978 |
|  | DM-TB | 1.275165 | 0.303068 | 4.208 | 0.0002 |
|  | DM-UM | 1.907724 | 0.350634 | 5.441 | <.0001 |
|  | DM-W | 2.605813 | 0.593823 | 4.388 | 0.0001 |
|  | RP-TB | 1.357063 | 0.307305 | 4.416 | 0.0001 |
|  | RP-UM | 1.989622 | 0.446915 | 4.452 | 0.0001 |
|  | RP-W | 2.687711 | 0.546246 | 4.92 | <.0001 |
|  | TB-UM | 0.632559 | 0.434243 | 1.457 | 0.5908 |
|  | TB-W | 1.330649 | 0.606724 | 2.193 | 0.1822 |
|  | UM-W | 0.698089 | 0.730038 | 0.956 | 0.8746 |
|  |  |  |  |  |  |
| 36 | DM-RP | -0.78251 | 0.290782 | -2.691 | 0.0553 |
|  | DM-TB | 0.349662 | 0.313897 | 1.114 | 0.7993 |
|  | DM-UM | 1.559274 | 0.41581 | 3.75 | 0.0017 |
|  | DM-W | 2.545086 | 0.787894 | 3.23 | 0.0109 |
|  | RP-TB | 1.132168 | 0.292454 | 3.871 | 0.001 |
|  | RP-UM | 2.34178 | 0.483838 | 4.84 | <.0001 |
|  | RP-W | 3.327592 | 0.742258 | 4.483 | 0.0001 |
|  | TB-UM | 1.209612 | 0.460591 | 2.626 | 0.0657 |
|  | TB-W | 2.195424 | 0.780953 | 2.811 | 0.0396 |
|  | UM-W | 0.985812 | 0.904435 | 1.09 | 0.8118 |
|  |  |  |  |  |  |
| 38 | DM-RP | -1.00285 | 0.295797 | -3.39 | 0.0063 |
|  | DM-TB | 0.762791 | 0.375778 | 2.03 | 0.2516 |
|  | DM-UM | 1.163818 | 0.392722 | 2.963 | 0.0253 |
|  | DM-W | 2.006491 | 0.67643 | 2.966 | 0.0251 |
|  | RP-TB | 1.765642 | 0.349624 | 5.05 | <.0001 |
|  | RP-UM | 2.166669 | 0.45767 | 4.734 | <.0001 |
|  | RP-W | 3.009342 | 0.617865 | 4.871 | <.0001 |
|  | TB-UM | 0.401027 | 0.476699 | 0.841 | 0.9177 |
|  | TB-W | 1.2437 | 0.693133 | 1.794 | 0.3768 |
|  | UM-W | 0.842673 | 0.791962 | 1.064 | 0.825 |
|  |  |  |  |  |  |
| 40 | DM-RP | -1.3397 | 0.319038 | -4.199 | 0.0003 |
|  | DM-TB | 0.115644 | 0.362328 | 0.319 | 0.9978 |
|  | DM-UM | 0.347219 | 0.372972 | 0.931 | 0.885 |
|  | DM-W | 1.669475 | 0.686868 | 2.431 | 0.1072 |
|  | RP-TB | 1.45534 | 0.31308 | 4.648 | <.0001 |
|  | RP-UM | 1.686915 | 0.424322 | 3.976 | 0.0007 |
|  | RP-W | 3.009171 | 0.617817 | 4.871 | <.0001 |
|  | TB-UM | 0.231575 | 0.416668 | 0.556 | 0.9813 |
|  | TB-W | 1.553831 | 0.675394 | 2.301 | 0.1446 |
|  | UM-W | 1.322256 | 0.773131 | 1.71 | 0.4275 |
|  |  |  |  |  |  |
| 42 | DM-RP | -1.05189 | 0.294465 | -3.572 | 0.0033 |
|  | DM-TB | 0.52155 | 0.348963 | 1.495 | 0.566 |
|  | DM-UM | 0.550187 | 0.344747 | 1.596 | 0.5 |
|  | DM-W | 16.52849 | 824.8837 | 0.02 | 1 |
|  | RP-TB | 1.573436 | 0.319376 | 4.927 | <.0001 |
|  | RP-UM | 1.602073 | 0.416268 | 3.849 | 0.0011 |
|  | RP-W | 17.58038 | 824.8837 | 0.021 | 1 |
|  | TB-UM | 0.028637 | 0.415225 | 0.069 | 1 |
|  | TB-W | 16.00694 | 824.8837 | 0.019 | 1 |
|  | UM-W | 15.97831 | 824.8838 | 0.019 | 1 |
|  |  |  |  |  |  |
| 44 | DM-RP | -1.15279 | 0.327766 | -3.517 | 0.004 |
|  | DM-TB | 0.253062 | 0.37968 | 0.667 | 0.9635 |
|  | DM-UM | 0.145017 | 0.368964 | 0.393 | 0.995 |
|  | DM-W | 0.922468 | 0.532097 | 1.734 | 0.4132 |
|  | RP-TB | 1.405849 | 0.333596 | 4.214 | 0.0002 |
|  | RP-UM | 1.297804 | 0.421185 | 3.081 | 0.0176 |
|  | RP-W | 2.075255 | 0.439842 | 4.718 | <.0001 |
|  | TB-UM | -0.10805 | 0.422217 | -0.256 | 0.9991 |
|  | TB-W | 0.669406 | 0.524481 | 1.276 | 0.7059 |
|  | UM-W | 0.777451 | 0.633092 | 1.228 | 0.735 |
|  |  |  |  |  |  |
| 46 | DM-RP | -0.52233 | 0.275144 | -1.898 | 0.3181 |
|  | DM-TB | 1.213404 | 0.359671 | 3.374 | 0.0067 |
|  | DM-UM | 1.343592 | 0.358138 | 3.752 | 0.0016 |
|  | DM-W | 3.660613 | 1.056665 | 3.464 | 0.0048 |
|  | RP-TB | 1.735735 | 0.349765 | 4.963 | <.0001 |
|  | RP-UM | 1.865923 | 0.442005 | 4.222 | 0.0002 |
|  | RP-W | 4.182944 | 1.02602 | 4.077 | 0.0004 |
|  | TB-UM | 0.130188 | 0.461705 | 0.282 | 0.9986 |
|  | TB-W | 2.447209 | 1.072972 | 2.281 | 0.151 |
|  | UM-W | 2.317021 | 1.133101 | 2.045 | 0.2446 |
|  |  |  |  |  |  |
| 48 | DM-RP | -0.99683 | 0.326566 | -3.052 | 0.0193 |
|  | DM-TB | 1.154067 | 0.480877 | 2.4 | 0.1153 |
|  | DM-UM | 0.679952 | 0.389202 | 1.747 | 0.405 |
|  | DM-W | 1.835552 | 0.687574 | 2.67 | 0.0585 |
|  | RP-TB | 2.150901 | 0.450255 | 4.777 | <.0001 |
|  | RP-UM | 1.676787 | 0.443862 | 3.778 | 0.0015 |
|  | RP-W | 2.832387 | 0.622414 | 4.551 | 0.0001 |
|  | TB-UM | -0.47411 | 0.534326 | -0.887 | 0.9017 |
|  | TB-W | 0.681485 | 0.746246 | 0.913 | 0.8919 |
|  | UM-W | 1.1556 | 0.781576 | 1.479 | 0.5765 |
|  |  |  |  |  |  |
| 50 | DM-RP | -0.71243 | 0.383947 | -1.856 | 0.3417 |
|  | DM-TB | 0.61836 | 0.459844 | 1.345 | 0.6631 |
|  | DM-UM | 2.22082 | 0.785691 | 2.827 | 0.0379 |
|  | DM-W | 2.085886 | 0.814747 | 2.56 | 0.0779 |
|  | RP-TB | 1.330795 | 0.424876 | 3.132 | 0.015 |
|  | RP-UM | 2.933255 | 0.812556 | 3.61 | 0.0028 |
|  | RP-W | 2.798321 | 0.758944 | 3.687 | 0.0021 |
|  | TB-UM | 1.60246 | 0.829185 | 1.933 | 0.2999 |
|  | TB-W | 1.467526 | 0.82765 | 1.773 | 0.3893 |
|  | UM-W | -0.13493 | 1.107051 | -0.122 | 1 |
|  |  |  |  |  |  |
| 52 | DM-RP | -0.79531 | 0.3537 | -2.249 | 0.162 |
|  | DM-TB | 1.282697 | 0.523139 | 2.452 | 0.1019 |
|  | DM-UM | 1.499332 | 0.55075 | 2.722 | 0.0508 |
|  | DM-W | 16.67581 | 948.056 | 0.018 | 1 |
|  | RP-TB | 2.078008 | 0.495646 | 4.193 | 0.0003 |
|  | RP-UM | 2.294643 | 0.590922 | 3.883 | 0.001 |
|  | RP-W | 17.47112 | 948.0559 | 0.018 | 1 |
|  | TB-UM | 0.216635 | 0.678985 | 0.319 | 0.9978 |
|  | TB-W | 15.39311 | 948.0561 | 0.016 | 1 |
|  | UM-W | 15.17648 | 948.0561 | 0.016 | 1 |
|  |  |  |  |  |  |
| 54 | DM-RP | -0.78865 | 0.396256 | -1.99 | 0.2707 |
|  | DM-TB | 2.577552 | 1.044381 | 2.468 | 0.098 |
|  | DM-UM | 0.380326 | 0.453645 | 0.838 | 0.9187 |
|  | DM-W | 2.667284 | 1.083615 | 2.461 | 0.0995 |
|  | RP-TB | 3.3662 | 1.028638 | 3.272 | 0.0094 |
|  | RP-UM | 1.168974 | 0.496829 | 2.353 | 0.1285 |
|  | RP-W | 3.455932 | 1.041448 | 3.318 | 0.0081 |
|  | TB-UM | -2.19723 | 1.069138 | -2.055 | 0.2399 |
|  | TB-W | 0.089731 | 1.43645 | 0.062 | 1 |
|  | UM-W | 2.286957 | 1.146146 | 1.995 | 0.2682 |
