# Supplementary material for: Escaping and repairing behaviors of the termite Odontotermes formosanus (Blattodea: Termitidae) in response to disturbance
Source: PeerJ. 2018 Mar 16;6:e4513. doi: 10.7717/peerj.4513 (PMC5858535; doi:10.7717/peerj.4513)
Supplement: Table S5 [file peerj-06-4513-s005.docx]

Table S5. Mixed-effect linear models were built to assess differences of termite escaping speed among different phases (periods), treating phase (periods) as a fixed factor and video as a random factor. Summary of post-hoc comparisons (Tukey’s Honestly Significant Difference tests) among speed of termites in the individual escaping phase (IE) and three periods of escaping flow phase (5-6 min after the escaping flow was formed [EF1], in the middle of the escaping flow duration [EF2], and 5-6 min before the escaping flow ended [EF3]) are shown.

| **Pairwise comparison** | **Estimate** | **SE** | **Z value** | ***P*** |
| --- | --- | --- | --- | --- |
| IE – EF1 | 0.898 | 0.034 | 36.721 | <0.0001 |
| IE – EF2 | 1.006 | 0.034 | 29.916 | <0.0001 |
| IE – EF3 | 1.028 | 0.034 | 30.575 | <0.0001 |
| EF1 – EF2 | 0.107 | 0.034 | 3.195 | 0.0075 |
| EF1 – EF3 | 0.130 | 0.034 | 3.854 | 0.0003 |
| EF2 – EF3 | 0.022 | 0.034 | 0.659 | 0.9123 |
