# Supplementary material for: Escaping and repairing behaviors of the termite Odontotermes formosanus (Blattodea: Termitidae) in response to disturbance
Source: PeerJ. 2018 Mar 16;6:e4513. doi: 10.7717/peerj.4513 (PMC5858535; doi:10.7717/peerj.4513)
Supplement: Table S6 [file peerj-06-4513-s006.docx]

Table S6: Summary of generalized linear mixed-effect models comparing the repairing area constructed from bottom to top with other directions of the termite (*Odontotermes formosanus*) after the mud tube had been damaged. The number following “time” indicates the time (minute) after tubes were damaged. “:” indicates the interaction between repairing direction and time

a) Damaged mud tubes were closed within 20 minute

|  | **Estimate** | **SE** | **t value** | ***P*** |
| --- | --- | --- | --- | --- |
| Intercept | 4.2002 | 0.1836 | 22.878 | <.0001 |
| top to bottom | -2.1454 | 1.59 | -1.349 | 0.177 |
| side | -0.7438 | 0.4248 | -1.751 | 0.08 |
| time10 | 1.0329 | 0.1796 | 5.751 | <.0001 |
| time15 | 1.5206 | 0.1731 | 8.787 | <.0001 |
| time20 | 1.6876 | 0.1749 | 9.649 | <.0001 |
| top to bottom:time10 | 0.2105 | 1.6539 | 0.127 | 0.899 |
| side:time10 | -0.2471 | 0.3774 | -0.655 | 0.513 |
| top to bottom:time15 | 0.7039 | 1.5999 | 0.44 | 0.66 |
| side:time15 | -0.4775 | 0.3696 | -1.292 | 0.196 |
| top to bottom:time20 | 0.804 | 1.6008 | 0.502 | 0.615 |
| side:time20 | -0.5316 | 0.3759 | -1.414 | 0.157 |

b) Damaged mud tubes were closed between 20 and 40 minute

|  | **Estimate** | **SE** | **t value** | ***P*** |
| --- | --- | --- | --- | --- |
| Intercept | 3.6276 | 0.1563 | 23.2050 | <.0001 |
| top to bottom | -1.7498 | 0.6640 | -2.6350 | 0.0084 |
| side | -0.9199 | 0.3400 | -2.7050 | 0.0068 |
| time10 | 0.7214 | 0.1369 | 5.2700 | 0.0000 |
| time15 | 1.2788 | 0.1278 | 10.0050 | <.0001 |
| time20 | 1.7263 | 0.1251 | 13.8020 | <.0001 |
| time25 | 2.0225 | 0.1242 | 16.2820 | <.0001 |
| time30 | 2.2632 | 0.1241 | 18.2370 | <.0001 |
| time35 | 2.5229 | 0.1241 | 20.3230 | <.0001 |
| time40 | 2.6449 | 0.1247 | 21.2130 | <.0001 |
| top to bottom:time10 | -0.1324 | 0.7388 | -0.1790 | 0.8578 |
| side:time10 | 0.2441 | 0.3521 | 0.6930 | 0.4880 |
| top to bottom:time15 | -0.0367 | 0.6738 | -0.0540 | 0.9565 |
| side:time15 | 0.0008 | 0.3396 | 0.0020 | 0.9980 |
| top to bottom:time20 | -0.1481 | 0.6612 | -0.2240 | 0.8228 |
| side:time20 | -0.2115 | 0.3350 | -0.6310 | 0.5277 |
| top to bottom:time25 | -0.0631 | 0.6542 | -0.0960 | 0.9232 |
| side:time25 | -0.3007 | 0.3329 | -0.9030 | 0.3663 |
| top to bottom:time30 | 0.0716 | 0.6516 | 0.1100 | 0.9125 |
| side:time30 | -0.5567 | 0.3330 | -1.6720 | 0.0945 |
| top to bottom:time35 | 0.1420 | 0.6535 | 0.2170 | 0.8279 |
| side:time35 | -1.0474 | 0.3379 | -3.1000 | 0.0019 |
| top to bottom:time40 | 0.4210 | 0.6585 | 0.6390 | 0.5226 |
| side:time40 | -1.0665 | 0.3436 | -3.1040 | 0.0019 |

c) Damaged mud tubes were closed beyond 40 minute

|  | **Estimate** | **SE** | **t value** | ***P*** |
| --- | --- | --- | --- | --- |
| Intercept | 0.0378 | 0.0079 | 4.7610 | <.0001 |
| top to bottom | 2.1713 | 0.8861 | 2.4500 | 0.0143 |
| side | 5.8489 | 1.3118 | 4.4590 | <.0001 |
| time15 | -0.0121 | 0.0068 | -1.7700 | 0.0767 |
| time20 | -0.0174 | 0.0063 | -2.7490 | 0.0060 |
| time25 | -0.0200 | 0.0062 | -3.2430 | 0.0012 |
| time30 | -0.0213 | 0.0061 | -3.4970 | 0.0005 |
| time35 | -0.0223 | 0.0060 | -3.6990 | 0.0002 |
| time40 | -0.0230 | 0.0060 | -3.8140 | 0.0001 |
| time50 | -0.0238 | 0.0060 | -3.9630 | 0.0001 |
| time55 | 0.0552 | 0.0179 | 3.0830 | 0.0020 |
| time60 | -0.0246 | 0.0060 | -4.0760 | <.0001 |
| top to bottom:time15 | -0.0389 | 0.0349 | -1.1140 | 0.2652 |
| side:time15 | -0.0627 | 0.0511 | -1.2260 | 0.2201 |
| top to bottom:time20 | -0.0558 | 0.0323 | -1.7260 | 0.0843 |
| side:time20 | -0.0869 | 0.0477 | -1.8240 | 0.0681 |
| top to bottom:time25 | -0.0635 | 0.0315 | -2.0170 | 0.0437 |
| side:time25 | -0.0868 | 0.0474 | -1.8300 | 0.0672 |
| top to bottom:time30 | -0.0679 | 0.0311 | -2.1850 | 0.0289 |
| side:time30 | -0.0952 | 0.0467 | -2.0390 | 0.0414 |
| top to bottom:time35 | -0.0696 | 0.0309 | -2.2520 | 0.0243 |
| side:time35 | -0.1014 | 0.0463 | -2.1930 | 0.0283 |
| top to bottom:time40 | -0.0712 | 0.0308 | -2.3120 | 0.0208 |
| side:time40 | -0.1006 | 0.0463 | -2.1750 | 0.0296 |
| top to bottom:time50 | -0.0750 | 0.0306 | -2.4490 | 0.0143 |
| side:time50 | -0.1013 | 0.0462 | -2.1920 | 0.0284 |
| top to bottom:time55 | 0.2513 | 0.1102 | 2.2800 | 0.0226 |
| side:time55 | 0.9540 | 0.3468 | 2.7510 | 0.0059 |
| top to bottom:time60 | -0.0734 | 0.0307 | -2.3900 | 0.0169 |
| side:time60 | -0.0963 | 0.0469 | -2.0530 | 0.0401 |
