# Supplementary material for: Escaping and repairing behaviors of the termite Odontotermes formosanus (Blattodea: Termitidae) in response to disturbance
Source: PeerJ. 2018 Mar 16;6:e4513. doi: 10.7717/peerj.4513 (PMC5858535; doi:10.7717/peerj.4513)
Supplement: Table S7 [file peerj-06-4513-s007.docx]

Table S7. Generalized linear mixed-effect models were built to compare the repairing area from different directions. Summary of post-hoc comparisons (Tukey’s Honestly Significant Difference tests) among areas that were repaired from bottom to top (BT), top to bottom (TB), and side (S) at each time interval are shown. The number in the “Time intervals” column indicates the time (minute) after the mud tubes were damaged

a) Damaged tubes were closed within 20 minute

| **Time intervals** | **Pairwise comparison** | **Estimate** | **SE** | **Z ratio** | ***P*** |
| --- | --- | --- | --- | --- | --- |
| 5 | BT-TB | 2.1454 | 1.5899 | 1.349 | 0.3679 |
|  | BT-S | 0.7438 | 0.4248 | 1.751 | 0.1865 |
|  | TB-S | -1.4016 | 1.6179 | -0.866 | 0.6616 |
|  |  |  |  |  |  |
| 10 | BT-TB | 1.9349 | 0.4643 | 4.167 | 0.0001 |
|  | BT-S | 0.9909 | 0.2761 | 3.589 | 0.001 |
|  | TB-S | -0.944 | 0.4966 | -1.901 | 0.1385 |
|  |  |  |  |  |  |
| 15 | BT-TB | 1.4415 | 0.1455 | 9.902 | <.0001 |
|  | BT-S | 1.2213 | 0.2531 | 4.825 | <.0001 |
|  | TB-S | -0.2202 | 0.217 | -1.015 | 0.5675 |
|  |  |  |  |  |  |
| 20 | BT-TB | 1.3414 | 0.1767 | 7.591 | <.0001 |
|  | BT-S | 1.2754 | 0.2654 | 4.805 | <.0001 |
|  | TB-S | -0.066 | 0.2458 | -0.268 | 0.9611 |

b) Damaged tubes were closed between 20 and 40 minute

| **Time intervals** | **Pairwise comparison** | **Estimate** | **SE** | **Z ratio** | ***P*** |
| --- | --- | --- | --- | --- | --- |
| 5 | BT-TB | 1.7498 | 0.6640 | 2.6350 | 0.0229 |
|  | BT-S | 0.9199 | 0.3400 | 2.7050 | 0.0187 |
|  | TB-S | -0.8299 | 0.7175 | -1.1570 | 0.4792 |
|  |  |  |  |  |  |
| 10 | BT-TB | 1.8822 | 0.3844 | 4.8960 | <.0001 |
|  | BT-S | 0.6757 | 0.1593 | 4.2420 | 0.0001 |
|  | TB-S | -1.2065 | 0.3933 | -3.0680 | 0.0061 |
|  |  |  |  |  |  |
| 15 | BT-TB | 1.7865 | 0.2353 | 7.5910 | <.0001 |
|  | BT-S | 0.9191 | 0.1297 | 7.0860 | <.0001 |
|  | TB-S | -0.8675 | 0.2419 | -3.5870 | 0.0010 |
|  |  |  |  |  |  |
| 20 | BT-TB | 1.8979 | 0.1964 | 9.6610 | <.0001 |
|  | BT-S | 1.1314 | 0.1164 | 9.7230 | <.0001 |
|  | TB-S | -0.7665 | 0.1995 | -3.8410 | 0.0004 |
|  |  |  |  |  |  |
| 25 | BT-TB | 1.8129 | 0.1707 | 10.6170 | <.0001 |
|  | BT-S | 1.2206 | 0.1081 | 11.2900 | <.0001 |
|  | TB-S | -0.5922 | 0.1701 | -3.4820 | 0.0014 |
|  |  |  |  |  |  |
| 30 | BT-TB | 1.6782 | 0.1626 | 10.3230 | <.0001 |
|  | BT-S | 1.4766 | 0.1102 | 13.3990 | <.0001 |
|  | TB-S | -0.2016 | 0.1637 | -1.2320 | 0.4344 |
|  |  |  |  |  |  |
| 35 | BT-TB | 1.6078 | 0.1640 | 9.8030 | <.0001 |
|  | BT-S | 1.9673 | 0.1257 | 15.6540 | <.0001 |
|  | TB-S | 0.3596 | 0.1757 | 2.0460 | 0.1014 |
|  |  |  |  |  |  |
| 40 | BT-TB | 1.3288 | 0.1765 | 7.5300 | <.0001 |
|  | BT-S | 1.9864 | 0.1404 | 14.1520 | <.0001 |
|  | TB-S | 0.6576 | 0.1973 | 3.3330 | 0.0025 |

c) Damaged tubes were closed beyond 40 minute

| **Time intervals** | **Pairwise comparison** | **Estimate** | **SE** | **Z ratio** | ***P*** |
| --- | --- | --- | --- | --- | --- |
| 10 | BT-TB | -2.1713 | 0.8861 | -2.4500 | 0.0379 |
|  | BT-S | -5.8489 | 1.3118 | -4.4590 | <.0001 |
|  | TB-S | -3.6776 | 1.4101 | -2.6080 | 0.0247 |
|  |  |  |  |  |  |
| 15 | BT-TB | -2.1324 | 0.8857 | -2.4070 | 0.0425 |
|  | BT-S | -5.7862 | 1.3112 | -4.4130 | <.0001 |
|  | TB-S | -3.6538 | 1.4094 | -2.5920 | 0.0258 |
|  |  |  |  |  |  |
| 20 | BT-TB | -2.1154 | 0.8857 | -2.3890 | 0.0446 |
|  | BT-S | -5.7619 | 1.3111 | -4.3950 | <.0001 |
|  | TB-S | -3.6465 | 1.4093 | -2.5880 | 0.0262 |
|  |  |  |  |  |  |
| 25 | BT-TB | -2.1078 | 0.8856 | -2.3800 | 0.0456 |
|  | BT-S | -5.7621 | 1.3111 | -4.3950 | <.0001 |
|  | TB-S | -3.6543 | 1.4093 | -2.5930 | 0.0258 |
|  |  |  |  |  |  |
| 30 | BT-TB | -2.1034 | 0.8856 | -2.3750 | 0.0462 |
|  | BT-S | -5.7537 | 1.3111 | -4.3880 | <.0001 |
|  | TB-S | -3.6503 | 1.4092 | -2.5900 | 0.0260 |
|  |  |  |  |  |  |
| 35 | BT-TB | -2.1016 | 0.8856 | -2.3730 | 0.0464 |
|  | BT-S | -5.7474 | 1.3111 | -4.3840 | <.0001 |
|  | TB-S | -3.6458 | 1.4092 | -2.5870 | 0.0262 |
|  |  |  |  |  |  |
| 40 | BT-TB | -2.1001 | 0.8856 | -2.3710 | 0.0466 |
|  | BT-S | -5.7482 | 1.3111 | -4.3840 | <.0001 |
|  | TB-S | -3.6482 | 1.4092 | -2.5890 | 0.0261 |
|  |  |  |  |  |  |
| 50 | BT-TB | -2.0963 | 0.8856 | -2.3670 | 0.0471 |
|  | BT-S | -5.7476 | 1.3111 | -4.3840 | <.0001 |
|  | TB-S | -3.6513 | 1.4092 | -2.5910 | 0.0259 |
|  |  |  |  |  |  |
| 55 | BT-TB | -2.4226 | 0.8914 | -2.7180 | 0.0181 |
|  | BT-S | -6.8029 | 1.3504 | -5.0380 | <.0001 |
|  | TB-S | -4.3803 | 1.4493 | -3.0220 | 0.0071 |
|  |  |  |  |  |  |
| 60 | BT-TB | -2.0979 | 0.8856 | -2.3690 | 0.0469 |
|  | BT-S | -5.7526 | 1.3111 | -4.3880 | <.0001 |
|  | TB-S | -3.6547 | 1.4092 | -2.5930 | 0.0257 |
